# The Effect of Gap Junctional Coupling on the Spatiotemporal Patterns of Ca^2+^ Signals and the Harmonization of Ca^2+^-Related Cellular Responses

**DOI:** 10.1371/journal.pcbi.1005295

**Published:** 2016-12-27

**Authors:** Michaël Dougoud, Laura Vinckenbosch, Christian Mazza, Beat Schwaller, László Pecze

**Affiliations:** 1 Department of Mathematics, University of Fribourg, Fribourg, Switzerland; 2 University of Applied Sciences and Arts Western Switzerland // HES-SO, HEIG-VD, Yverdon-les-Bains, Switzerland; 3 Anatomy, Department of Medicine, University of Fribourg, Fribourg, Switzerland; George Mason University, UNITED STATES

## Abstract

Calcium ions (Ca^2+^) are important mediators of a great variety of cellular activities e.g. in response to an agonist activation of a receptor. The magnitude of a cellular response is often encoded by frequency modulation of Ca^2+^ oscillations and correlated with the stimulation intensity. The stimulation intensity highly depends on the sensitivity of a cell to a certain agonist. In some cases, it is essential that neighboring cells produce a similar and synchronized response to an agonist despite their different sensitivity. In order to decipher the presumed function of Ca^2+^ waves spreading among connecting cells, a mathematical model was developed. This model allows to numerically modifying the connectivity probability between neighboring cells, the permeability of gap junctions and the individual sensitivity of cells to an agonist. Here, we show numerically that strong gap junctional coupling between neighbors ensures an equilibrated response to agonist stimulation via formation of Ca^2+^ phase waves, i.e. a less sensitive neighbor will produce the same or similar Ca^2+^ signal as its highly sensitive neighbor. The most sensitive cells within an ensemble are the wave initiator cells. The Ca^2+^ wave in the cytoplasm is driven by a sensitization wave front in the endoplasmic reticulum. The wave velocity is proportional to the cellular sensitivity and to the strength of the coupling. The waves can form different patterns including circular rings and spirals. The observed pattern depends on the strength of noise, gap junctional permeability and the connectivity probability between neighboring cells. Our simulations reveal that one highly sensitive region gradually takes the lead within the entire noisy system by generating directed circular phase waves originating from this region.

## Author Summary

The calcium ion (Ca^2+^), a universal signaling molecule, is widely recognized to play a fundamental role in the regulation of various biological processes. Agonist–evoked Ca^2+^ signals often manifest as rhythmic changes in the cytosolic free Ca^2+^ concentration (c_cyt_) called Ca^2+^ oscillations. Stimuli intensity was found to be proportional to the oscillation frequency and the evoked down-steam cellular response. Stochastic receptor expression in individual cells in a cell population inevitably leads to individually different oscillation frequencies and individually different Ca^2+^-related cellular responses. However, in many organs, the neighboring cells have to overcome their individually different sensitivity and produce a synchronized response. Gap junctions are integral membrane structures that enable the direct cytoplasmic exchange of Ca^2+^ ions and InsP_3_ molecules between neighboring cells. By simulations, we were able to demonstrate how the strength of intercellular gap junctional coupling in relation to stimulus intensity can modify the spatiotemporal patterns of Ca^2+^ signals and harmonize the Ca^2+^-related cellular responses via synchronization of oscillation frequency. We demonstrate that the most sensitive cells are the wave initiator cells and that a highly sensitive region plays an important role in the determination of the Ca^2+^ phase wave direction. This sensitive region will then also progressively determine the global behavior of the entire system.

## Introduction

Calcium ions (Ca^2+^) play a crucial role for almost every aspect in the biology of organisms. Cells possess sophisticated machinery to precisely regulate the free Ca^2+^ concentrations in the cytoplasm (c_cyt_), the endoplasmic reticulum (c_ER_) and the mitochondria (c_mito_). Maintaining the low concentrations of Ca^2+^ in the cytoplasm against a 10,000-fold higher extracellular Ca^2+^ concentration, i.e. the strong trans-membrane electrochemical gradient of Ca^2+^ ions needed for proper cell signaling [1] requires energy. Upon agonist stimulation, cytoplasmic Ca^2+^ levels are elevated from two sources: (i) Ca^2+^ influx from the extracellular space across the plasma membrane and (ii) Ca^2+^ release from stores, mostly the endoplasmic reticulum (ER). Different types of Ca^2+^ channels are responsible for the Ca^2+^ influx across the plasma membrane including: voltage-dependent Ca^2+^ channels, receptor-operated Ca^2+^ channels including transient receptor potential channels (TRP), store-operated Ca^2+^ channels, etc. [2]. The release of Ca^2+^ from the ER results from activation of either the ryanodine receptors (RyR) or the inositol 1,4,5-trisphosphate (InsP_3_) receptors (InsP_3_R). Previously, it was assumed that RyR are of primary importance for Ca^2+^ release in excitable cells, while InsP_3_R were presumed to govern Ca^2+^ release in non-excitable cells. However, both InsP_3_R and RyR are expressed in excitable as well as in non-excitable cells [3,4], indicating cooperation between the two types of receptors in all cell lines. RyR have structural and functional similarities with InsP_3_R, but show no sensitivity to InsP_3_ [5]. One of the roles of RyR is to amplify the InsP_3_-mediated release of Ca^2+^ [6]. Ca^2+^ signals are often organized in specific temporal patterns. The rhythmic changes in c_cyt_ are called Ca^2+^ oscillations. Several ligand/receptor interactions together with the involvement of components of the intracellular Ca^2+^-signaling toolkit induce Ca^2+^ oscillations [7,8] and as a result Ca^2+^ oscillations can act as integrators of different stimuli [9]. The stimuli intensity was often found to be proportional to the oscillation frequency, which in turn was proportional to the evoked down-steam cellular response, e.g. histamine-dependent fluid secretion of blowfly salivary gland [10] or glucose-dependent insulin secretion of pancreatic islets of Langerhans [11].

An identical genotype, differentiation state and moreover a stable environment are not sufficient to guarantee the same phenotype for neighboring cells within a tissue or organ. Indeed, single cell analysis of genetically identical cells grown *in vitro* revealed rather large cell-to-cell variability [12]. The transcription of DNA to mRNA followed by translation to protein occurs stochastically, as a consequence of the low copy number of DNA and mRNA molecules involved [13–15]. Therefore, each cell is expected to have a stochastic number of receptors for a certain ligand or agonist. This leads to an individually different sensitivity to agonists and individually different cellular response. For instance, Ca^2+^ responses in individual mesothelial cells show a wide range of different oscillatory patterns within a genetically homogenous cell population [16]. Nevertheless, in many organs, the neighboring cells have to overcome their individually different sensitivity and produce a synchronized response for instance, smooth muscle cells, in order to generate the contractile waves of the uterus or the peristaltic movement in the gastrointestinal tract.

Gap junctions are integral membrane structures that enable the direct exchange of cytoplasmic constituents (ions and low molecular weight metabolites) between neighboring cells. The core proteins of these channels are the connexins [17]. Gap junctions are permeable to small molecules including both Ca^2+^ and InsP_3_. Thus, gap junctions are involved in intercellular Ca^2+^ signaling. Besides forming gap junctions between the same cell types, numerous gap junctions are also known to exist between different cell types. For example, gap junction channels ensure heterocellular Ca^2+^ waves between glia and neurons [18]. Intercellular Ca^2+^ waves spreading via gap junctions occur in rat liver epithelial cells upon mechanical stimulation [19]. Besides of gap junctional transport of Ca^2+^ and/or InsP_3_, ATP may serve as a coupling messenger. ATP is thought to be released into the extracellular space and subsequently activating adjacent cells through purinergic receptors [20]. The ATP-mediated Ca^2+^ spreading within cell populations is slower than direct gap junctional coupling, but allows a connection between cells not connected by gap junctions [19].

Two types of Ca^2+^ waves can be distinguished associated with gap junctional coupling: i) Ca^2+^ “diffusion” or trigger waves and ii) Ca^2+^ phase waves. Ca^2+^ trigger waves arise, when a single cell is stimulated in the network. In this case the gap junctional transport of InsP_3_ originating from the stimulated cell drives the Ca^2+^ wave. In this case, local increases in c_cyt_ may be considered as an indicator of intercellular diffusion of InsP_3_ molecules and not of the bulk movement of Ca^2+^ ions [21]. Ca^2+^ trigger waves are slow and due to the dilution of InsP_3_, the intensities of the Ca^2+^ signal decrease in distant cells and finally Ca^2+^ waves fade out. In the case of Ca^2+^ phase waves, all cells are stimulated in the network, yet to different extents. Ca^2+^ phase waves are generated by a small shift in the phase between individual cells oscillating with the same or nearly the same frequencies. Evidently, a coupling agent is required that synchronizes the ensemble of cells. A Ca^2+^ phase wave differs from a trigger wave, since the spreading of Ca^2+^ phase waves can be much faster than diffusion and moreover can travel long distances without annihilation [22].

Many different models have been built to understand the versatile patterns of travelling Ca^2+^ waves [23,24]. However, these models do not take into account the heterogeneous sensitivity of cells to stimulation by agonists. This heterogeneity is assumed to substantially contribute to different individual temporal patterns observed in physiological settings. In this article, we address this issue. In order to decipher the presumed function of Ca^2+^ waves spreading among neighboring cells, a general, not cell-type specific mathematical model was developed. The concept of this general approach is based on the hypothesis of Fewtrell [25]: “Since a single cell type may exhibit most, if not all, of the different types of oscillatory patterns, it seems unlikely that each cell type has developed its own specific mechanism for the generation of Ca^2+^ oscillations. Instead, each cell may either contain a number of different mechanisms or a single, rather complex mechanism that is capable of generating the full range of oscillatory patterns”. Our model, although containing several simplifications related to the machinery implicated in Ca^2+^ oscillations, allows to numerically modifying (i) the connectivity probability between neighboring cells, (ii) the permeability of gap junctions and (iii) the sensitivity of a single cell to a particular agonist. The intercellular heterogeneity in agonist sensitivity can manifest in the onset of a Ca^2+^ response, as alterations in the steady-state levels of InsP_3_ and/or changes in the Ca^2+^ handling. The main goal of this work is thus to provide a coherent model for intercellular Ca^2+^ waves propagation and analyze the effects of gap junctions on networks of cells with inhomogeneous properties. This model highlights possible situations leading to the formation of typical signaling wave patterns (such as rings or spirals). In order to conduct our investigations, we analyze them in a first step within a deterministic framework, where the parameter values on the network are fixed. In such a manner, prominent parameters can be isolated. In a second step, noise is added to the system to stick to more realistic situations and its influence is investigated.

## Materials and Methods

### Cell network and random graph

We generalize the model of Pecze and Schwaller [16,26] by embedding it into a network of *N* = *n m* cells on a two-dimensional graph of size n × m. Each cell *v*_*ij*_, 1 ≤ *i* ≤ *n*, 1 ≤ *j* ≤ *m* is composed of the endoplasmic reticulum (ER) and the cytosol and whose dynamics is similar as the one depicted in [16]. We consider that cytosolic Ca^2+^ ions can pass from one cell to another via gap junction [17], but that ER lumen from neighboring cells are not connected, thus not allowing direct transfer of ER luminal Ca^2+^. The parameter *d* denotes the strength of gap junctional coupling; the stronger the gap junctional coupling, the faster the diffusion of Ca^2+^ ions between linked cells. For simplicity, only the diffusion of Ca^2+^ ions is considered in the models reported in the Main Text, in line with the study of Hofer [27] or Harris and Timofeeva [28]. In the supplemental S1 Text, we extend these models by also considering InsP_3_ diffusion through gap junction and show that InsP_3_ can also function as the molecule involved in synchronization of Ca^2+^ oscillation in cell types, where Ca^2+^ spikes are connected to InsP_3_ fluctuations [29].

Synchronization means here that two randomly selected neighboring cells tend to adjust the times at which they produce Ca^2+^ peaks (not the amplitude of these peaks). This is known as phase-synchronization [30,31]. First we select two neighboring cells in the network, *u* ~ *v* and estimate the phases of the time series *X*_*u*_ and *X*_*v*_ (Ca^2+^ concentrations within cytosol) using the interpolation technique described in [32]. Denote by τ_1_, τ_2_, … the times at which *X*_*u*_ attain its maxima. Between two maxima, the phase increases by 2*π* and in between a linear interpolation is used, so that the phase of *X*_*u*_ for τ_n_ ≤ *t* < τ_*n*+1_ is defined by

$$\Phi_{u}\left(t\right)\;=\;2\pi \left(\frac{t-\tau_{n}}{\tau_{n+1}-\tau_{n}}+n\;-\;1\right)$$ (1)

We consider now the differences of the phase of *X*_*u*_(*t*) and *X*_*v*_(t) and rescale these angles in the interval [0, 2*π*]

$$\Delta \phi_{u,v}\left(t\right)=\left(\Phi_{u}\left(t\right)\;-\;\Phi_{v}\left(t\right)\right)\;\text{ mod}\;\;2\pi$$ (2)

In a phase-locked situation, this difference would always be the same over time and would result in a constant. If this constant is 0, it means that *X*_*u*_(*t*) and *X*_*v*_(*t*) are perfectly synchronized, that is, all their peaks occur simultaneously. When the phases vary over time, so do their differences. Therefore, in order to quantify synchronization, one has to consider the distribution over time of the phase differences. The more the distribution is concentrated around 0, the more synchronized *X*_*u*_(*t*) and *X*_*v*_(*t*) are. A uniform distribution corresponds to the null case of no synchronization.

To quantify the concentration of the phase differences distribution Tass et al. [30] and Cazelles and Stone [32] use the following synchronization index, based on the Shannon entropy,

$$Q_{u,v}\;=\;\frac{S_{max,u,v}\;-\;S_{u,v}}{S_{max,u,v}}$$ (3)

where the Shannon entropy *S*_*u*, *v*_ is estimated by $-\Sigma_{k=1}^{n_{b}\left(u,v\right)}p_{k}\left(u,v\right)\text{log}p_{k}\left(u,v\right)$ and the maximal Shannon entropy is *S*_*max*,*u*,*v*_ = log(*n*_*b*_(*u*, *v*)). The number of class intervals is *n*_*b*_(*u*, *v*) and *p*_*k*_(*u*, *v*) denotes the relative frequency that Δ*ϕ*_*u*,*v*_ lies in the *k*^th^ interval. The index *Q*_*u*,*v*_ lies between 0 (no phase-synchronization) and 1 (perfect synchronization). Finally, we define a synchronization measure of the network by taking the mean of *Q*_*u*,*v*_ over all neighboring cells *u* ~ *v*

$$m_{sync}=\frac{1}{\left|E\right|}\sum_{u\sim v}\;Q_{u,v}$$ (4)

where |*ε*| is the number of edges, i.e. pairs of neighbors in the network. When *m*_*sync*_ = 1, the synchronization between all cells is perfect and in particular no wave nor any pattern would appear. For medium ranges of *m*_*sync*_, synchronization between neighboring cell is efficient enough to enable waves to emerge. When *m*_*sync*_ is close to zero, all cells tend to behave independently from each other. Our synchronization measure *m*_*sync*_ indicates the degree of phase synchrony focusing on time periods at which peaks are produced within the whole network. Amplitudes of the peaks are not taken into account for the parameter *m*_*sync*_.

**Fig 1:**
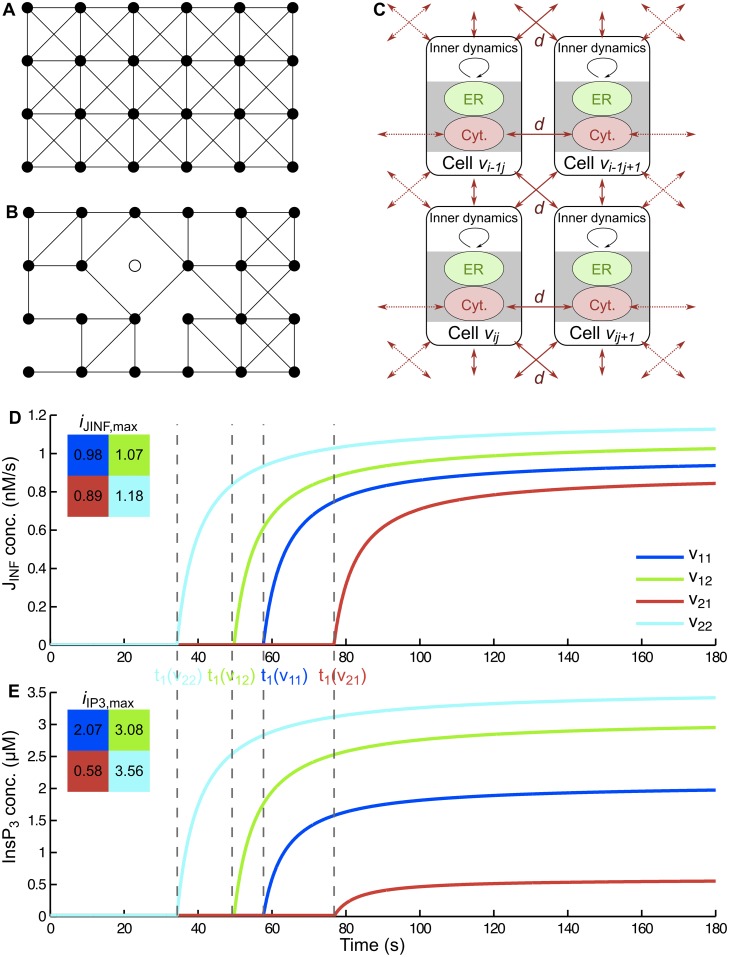
Cell networks. (A) The graph $G_{0}$ with *n* = 4 and *m* = 6. (B) A random version of it. Each link from $G_{0}$ was removed with a given probability *p*(*v*). Holes are allowed by choosing some threshold for removing nodes. (C) Each cell contains two components: the cytoplasmic compartment (Cyt) and the endoplasmic reticulum (ER). Neighboring cells exchange Ca^2+^ ions through their cytoplasmic compartments defined by the gap junctional coupling parameter *d*. (D-E) Graphical representation of *J*_*INF*_(*v*, *t*) (D) and *InsP*_3_(*v*, *t*) (E) in a simple inhomogeneous 2 × 2 grid with cells label by *v*_11_, *v*_12_, *v*_21_, *v*_22_. See Eqs (20) and (21). The parameter values of *i*_*JINF*,*max*_(*v*), *i*_*IP3*,*max*_(*v*) and *t*_1_(*v*) are represented graphically in order to illustrate our correlations assumptions.

Two graph topologies were used: homogeneous, fully dense graph and heterogeneous sparse graph topologies (Fig 1).

I) The homogeneous, fully dense graph topology consists of a grid graph to which diagonals were added ($G_{0}$, Fig 1A). In this setting, all neighboring cells are connected to each other. The set of nodes is *V*_0_ = {*v*_*ij*_, 1 ≤ *i* ≤ *n*, 1 ≤ *j* ≤ *m*} and let *ε*_0_ denote the set of links. Let $v_{i_{1}j_{1}}$ and $v_{i_{2}j_{2}}$ be two members of set *V*_0_. These nodes are connected in $G_{0}$, if and only if they are neighbors, that is,

$$\left\{v_{i_{1}j_{1}},\;\;v_{i_{2}j_{2}}\;\right\}\;\in \;E_{0}\;\overset{}{\;\Leftrightarrow \;}\;{\left|i_{1}-i_{2}\right|}^{2}\;+\;\;{\left|j_{1}-j_{2}\right|}^{2}\;\leq \;2.$$ (5)

II) Heterogeneous sparse graph (G) is a random sparse version generated from the homogeneous fully dense graph ($G_{0}$). In this situation, some links and nodes were randomly removed, exemplified in Fig 1B. Decreased gap junction coupling can be achieved by decreasing the permeability or numbers of the specific channels involved [21,33]. The latter situation can be modeled by link removal. Changes in the densities of gap junctions (strong decrease and recovery) was observed after partial hepatectomy [34]. Random sparse graphs were obtained by the following procedure. The link-removal probability *p*(*v*) is built with a colored noise in space, in order to create spatial correlations among the network. Let *F* be a Gaussian random field on the graph with standard deviation set to one and with a correlation function of the Gaussian form, that is

$$Cor\left(F\left(x\right),F\left(y\right)\right)\propto \;\;exp\left(-\;\frac{∥x-y{∥}^{2}}{c_{0}}\right),\;\;\;\;\;x,y\in {\mathbb{R}}^{2},$$ (6)

where $c_{0}=\sqrt{n^{2}+m^{2}}$ is a correlation parameter. When randomized, the probabilities *p*(*v*) are as follows

$$p\left(v\right)=p\;\frac{max\left(F\right)-\;F\left(v\right)}{max\left(F\right)-min\left(F\right)}$$ (7)

for some *p* ∈ [0, 1] in a way that in regions with nodes having low values of *F*, the probability to remove links is high, creating sparse areas, whereas regions with large values of *F* tend to be more strongly linked. Choosing a small quantile *q* of *F*, it is possible to create holes in the network by excluding nodes *v* from *V*_0_ whenever *F*(*v*) < *q*. The resulting graph is denoted by G=(V, E), (Fig 1B).

### Dynamics of Ca^2+^ concentrations on the network

Denote by (*X*_*v*_(*t*), *Y*_*v*_(*t*)) the free Ca^2+^ concentrations in the cytosol (c_cyt_) and in the ER (c_ER_) of the cell *v* at time *t*. The corresponding dynamical system on the graph G is driven by the following system of differential equations:

$$\{\begin{array}{l} \frac{dX_{v}}{dt}\;=\;J_{INF}\left(v,t\right)+f\left(X_{v},Y_{v},v,t\right)+d\underset{\left\{v,u\right\}\in E}{\sum }\left(X_{u}-X_{v}\right)\\ \frac{dY_{v}}{dt}\;=\;g\left(X_{v},Y_{v},v,t\right), \end{array}$$ (8)

for all *v* ∈ *V* with initial conditions *X*_*v*_(0) = c_*cyt*,*ini*_ = 110nM and *Y*_*v*_(0) = c_*ER*,*ini*_ = 260μM according to [16]. At the boundaries or in the case of holes, fluxes follow only existing directions, and there are no fluxes via the missing links. The function *J*_*INF*_(*v*,*t*) comprises the following movements of Ca^2+^ ions that increase the free Ca^2+^ concentration in the cytoplasmic compartment: (i) Ca^2+^ ions entering the cytoplasm from the extracellular space or from organelles (mitochondria, Golgi apparatus, lysosomes) and (ii) Ca^2+^ ions released from cytoplasmic Ca^2+^-binding proteins, which are considered as Ca^2+^ buffers in a broad sense. In a stricter sense, Ca^2+^ buffers comprise specific subsets of mobile Ca^2+^-binding proteins such as calretinin, calbindin D-28k and parvalbumin. The particular case of calretinin is presented in the S2 Text). The diffusion of Ca^2+^ through gap junctions from a cell’s cytosol to the one of an adjacent cell is given by the term *d* Σ_{*v*,*u*}∈ε_(*X*_*u*_ − *X*_*v*_), with *d* the gap junctional coupling strength. The functions *f* and *g* of concentrations *x*, *y* regulate the Ca^2+^ exchanges between cytosol and ER and are defined by

$$f\left(x,y,v,t\right)=\;\;-J_{EFF}\left(x\right)-J_{SERCA}\left(x\right)+J_{EREFF}\left(x,y,v,t\right)+J_{ERLEAK}$$ (9)

$$g\left(x,y,v,t\right)=\;\gamma \left(J_{SERCA}\left(x\right)-J_{EREFF}\left(x,y,v,t\right)-J_{ERLEAK}\right),$$ (10)

where the constant *γ* is the ratio between the changes in *X*_*v*_ and *Y*_*v*_ caused by the same amount of Ca^2+^ ions transported through the ER membrane. This value is derived from the difference in the effective volume of the ER lumen and the cytosolic volume and from the different fractions of free and protein-bound Ca^2+^ in these compartments. The function J_EFF_ represents a combination of Ca^2+^ fluxes: (i) from the cytosol to the extracellular space, (ii) from cytosol to organelles and (iii) Ca^2+^ ions temporarily sequestered by cytosolic Ca^2+^ buffers. The common features of these Ca^2+^-ion movements are their dependence on c_cyt_. The higher c_cyt_ is, the more Ca^2+^ ions are removed by mitochondria or plasma membrane extrusion systems, or are bound to cytosolic Ca^2+^ buffers. We simulated J_EFF_ by a linear equation, in line with the work of Fink et al [35] and based on the experimental results of Herrington et al. [36]. The different dissociation constant (K_d_) values of individual components (plasma membrane Ca^2+^ ATPase’s, exchangers, mitochondria) ensure that the extrusion fluxes will never reach their saturation points in the range of biologically relevant values of c_cyt_.

$$J_{EFF}\left(x\right)=\left(r_{e_{1}}x-r_{e_{2}}\right)1_{\left\{r_{e_{1}}x-r_{e_{2}}>0\right\}}$$ (11)

where the indicator function **1**_{*x*>0}_ returns 1 if *x* > 0 and 0 otherwise. The function *J*_*SERCA*_ denotes the Ca^2+^ flux from the cytosol to the ER. For simplicity, we assume that it depends only on c_cyt_, in line with our previous study [16]. Here, we also assumed that the different SERCA variants with different K_d_ values [37] ensure that this flux will never reach its saturation point in the range of biologically relevant values of c_cyt_. It is given by

$$J_{SERCA}\left(x\right)=\left(r_{s_{1}}x-r_{s_{2}}\right)1_{\left\{r_{s_{1}}x-r_{s_{2}}>0\right\}}$$ (12)

Modeling of *J*_*SERCA*_ and *J*_*EFF*_ fluxes with saturating Hill equations, did not modify qualitatively the behavior of our model. The simulations are presented in S3 Text.

The parameter *J*_*ERLEAK*_ represents a small flux of Ca^2+^ ions diffusing from the ER to the cytosol. The origin of this flux is unknown as well as its main characteristics [38]. For simplicity, we considered this flux to be constant,

$$J_{ERLEAK}=\beta$$ (13)

*J*_*EREFF*_ describes the flux of Ca^2+^ passing from the ER to the cytosol through InsP_3_R. In our model, InsP_3_R are influenced both by c_cyt_ and by c_ER_, however without an allosteric regulation between the two. We consider *J*_*EREFF*_ as the sum of two individual functions. The first one depends on c_cyt_ and has a bell-shaped form when considering concentrations in logarithmic scale [39],

$$J_{cytdep}\left(x,v,t\right)=\;R_{i,max}\left(v,t\right)exp\left(-\frac{{\left(\text{log}\left(x\right)-M\left(v,t\right)\right)}^{2}}{\sigma^{2}}\right)$$ (14)

where *M* is the concentration mean on a logarithmic scale, *R*_*i*,*max*_ the maximum and where the variance σ^2^ is a positive constant. The second function expresses the experimental fact that an increase in [InsP_3_] causes significant Ca^2+^ release from the ER even if c_cyt_ = 0 [40].

$$J_{ERdep}\left(y,v,t\right)=\;\;r_{i,1}\;log\left(y\right)-R_{i,2}\left(v,t\right)$$ (15)

where *r*_*i*,1_ and *R*_*i*,2_ are positive. We thus define

$$\begin{array}{l} J_{EREFF}\left(x,y,v,t\right)\\ =\;\left(J_{cytdep}\left(x,v,t\right)+J_{ERdep}\left(y,v,t\right)\right)\;\cdot \;1_{\left\{J_{cytdep}\left(x,v,t\right)+J_{ERdep}\left(y,v,t\right)>0\right\}} \end{array}$$ (16)

The shape of *J*_*EREFF*_ depends on [InsP_3_] and is encapsulated in the functions *M*, *R*_*i*,*max*_ and *R*_*i*2_. Indeed, [InsP_3_] modifies the sensitivity of InsP_3_R to changes in c_cyt_ and in c_ER_. Elevating [InsP_3_] mainly changes the mean (*M* on a logarithmic scale) and the maximum (*R*_*i*,*max*_) of the bell-shaped curve of c_cyt_ dependence [41]. In our model, we did not consider that the open probability curve at high [InsP_3_] is not precisely bell-shaped [41]. For simplicity, we didn’t consider that the Ca^2+^ flux from the ER, either through leak channels or InsP_3_R depends on the driving force originating from an electrochemical gradient across the ER membrane. W**e** used stationary open probabilities, not considering the binding/unbinding kinetics of Ca^2+^ to activating or inhibitory sites of InsP_3_R. Taking the abovementioned factors into account did not modify critically the behavior of the system. The simulations are presented in S3 Text. Based on the experimental data presented in [42,43], elevating [InsP_3_] also has an effect on the loading of the ER. Increased [InsP_3_] reduces the amount of Ca^2+^ ions stored in the ER. We simulated this effect by changing the parameter *R*_*i*2_. The functions *M*, *R*_*i*,*max*_ and *R*_*i*,2_ have similar forms, given by

$$M\left(v,t\right)=\mu_{min}+\left(\mu_{max}-\mu_{min}\right)\frac{K_{b}}{K_{b}+InsP_{3}\left(v,t\right)}$$ (17)

$$R_{i,max}\left(v,t\right)\;=\;r_{im,min}\;+\;\left(r_{im,max}\;-\;r_{im,min}\right)\;\frac{K_{b}}{K_{b}+InsP_{3}\left(v,t\right)}$$ (18)

$$R_{i,2}\left(v,t\right)\;=\;r_{i2,min}\;+\;\left(r_{i2,max}\;-\;r_{i2,min}\right)\;\frac{K_{b}}{K_{b}+InsP_{3}\left(v,t\right)}$$ (19)

We assume that within cell populations, each cell has a different sensitivity to agonists. A stimulation induces two processes that are important for Ca^2+^ oscillations: (i) it increases the levels of InsP_3_ by G-protein-regulated phospholipase C and (ii) it increases the Ca^2+^ flux characterized by *J*_*INF*_, mainly due to the opening of plasma membrane Ca^2+^ channels. We assume that both processes are positively related to the stimulus intensity, but two cells with the same [InsP_3_] can have different *J*_*INF*_ values. Hence, we used two input parameters:

$$InsP_{3}\left(v,t\right)=\{\begin{array}{rr} 0.015\;, & \text{if}\;t<t_{1}\left(v\right)\\ i_{IP3,max}\left(v\right)\frac{t-t_{1}\left(v\right)}{K_{IP3}+\left(t-t_{1}\left(v\right)\right)}, & \text{if}\;t\geq t_{1}\left(v\right) \end{array}$$ (20)

and

$$J_{INF}\left(v,t\right)=\;\{\begin{array}{rr} 0.1\;, & \text{if}\;t<t_{1}\left(v\right)\\ i_{JINF,max}\left(v\right)\frac{t-t_{1}\left(v\right)}{K_{JINF}+\left(t-t_{1}\left(v\right)\right)}, & \text{if}\;t\geq t_{1}\left(v\right) \end{array}$$ (21)

where *t*_1_ is the time point of the onset of increase in intracellular InsP_3_ levels and of the increase of the Ca^2+^ flux characterized by *J*_*INF*_. *K*_*IP*3_ and *K*_*JINF*_ are positive constants. The functions *InsP*_3_(*v*, *t*) and *J*_INF_(*v*, *t*) can be inhomogeneous in space, hence depending on the cell *v*. Initially, they are set to small values, which are constant in time and identical for all cells. At time *t*_1_(*v*), the processes start and both functions rapidly increase to approach limiting values *i*_*IP*3,*max*_(*v*) and *i*_*JINF*,*max*_(*v*), respectively. This leads to different frequencies of single cell Ca^2+^ oscillations. In accordance with experiments [44], we assumed that stronger activation results in smaller *t*_1_ value. Thus, the values of *i*_*JINF*,*max*_(*v*) and *i*_*IP*3_,_*max*_(*v*) are positively correlated among cells, but the values of *i*_*JINF*,*max*_(*v*) and *t*_1_(*v*) are negatively correlated, as exemplified in Fig 1D and 1E. In our model, we did not consider that J_INF_ is partially sensitive to changes in c_cyt_. All parameter values are presented in Table 1. All these values were set to reproduce as closely as possible the Ca^2+^ concentration changes in cytosol and ER measured experimentally by fluorescent Ca^2+^ indicators.

**Table 1:** Parameters. See [16] for more details.

	Parameter name	Value		
Constants	*γ*	450		
	$\left(r_{e_{1}},\;r_{e_{2}}\right)$	0.17 /s, 18.8 nM/s		
	$\left(r_{s_{1}},\;r_{s_{2}}\right)$	0.27 /s, 26.5 nM/s		
	*σ*	0.1 nM		
	*r*_*i*,1_	1300 /s		
	(μ_*min*_, μ_*max*_)	(2.4, 2.18) nM		
	(*r*_*im*,*min*_, *r*_*im*,*max*_)	(821.3, 24.3) nM/s		
	(*r*_*i*2,*min*_, *r*_*i*2,*max*_)	(6352, 7042) nM/s		
	*K*_*b*_	1 μM		
	*β*	2.5 nM/s		
	*X*_*v*_(0), *Y*_*v*_(0)	110 nM, 260 μM		
Influx and sensitivity	*i*_*IP*3,*max*,0_, $\mu_{i_{IP3,max}}$	1.8 μM, 1.8 μM		
	*i*_*JINF*,*max*,0_, $\;\mu_{i_{JINF,max}}$	0.9 nM/s, 0.9 nM/s		
	*t*_1,0_, $\mu_{t_{1}}$	60 s, 60 s		
	*K*_*IP*3_, *K*_*JINF*_	6 s, 6 s		
	$\left(\sigma_{i_{IP3,max}},\;\sigma_{i_{JINF,max}},\sigma_{t_{1}}\right)$	Low	Moderate	High
		(0.2, 0.01, 4)	(0.4, 0.025, 10)	(0.6, 0.04, 10)
Graph and Coupling	(*n*, *m*)	(25, 25)		
	$\left(\tilde{n},\tilde{m}\right)$	(3, 3)		
	*d*	Low	Moderate	High
		0.0015	0.0045	0.0075

### Particular models

We present and analyze four particular types of models and networks, each type allowing to highlight different phenomena and to isolate the key features leading to the various patterns arising.

#### Model G_0_: Individual cell

Oscillations in a single cell are investigated, shedding light on the parameter values leading to the oscillatory response to an input signal. The parameters are fixed according to Table 1 unless specified.

#### Model G_D_: Noise-free graph with distinct regions

We consider the graph $G_{0}$. All parameters are deterministic and fixed for all nodes *v* ∈ *V*_0_. Onto this network, we introduce 1, 2 or 3 regions with different sensitivities and inputs. More formally, we define s∈ℕ subgraphs $\left({\tilde{G}}_{j}\right){\;}_{1,\ldots ,s}$ of sizes ${\tilde{n}}_{j}\;\times \;{\tilde{m}}_{j}$, within the graph $G_{0}$. The subgraphs are disjoint (i.e. with no common nodes). Thus *i*_*JINF*,*max*_(*v*) takes the form:

$$i_{JINF,max}\left(v\right)\;=\;\{\begin{array}{ll} i_{JINF,max,0}, & \text{if}\;v\in V_{0}\backslash \cup_{j=1}^{s}{\tilde{V}}_{j}\\ i_{JINF,max,1}, & \text{if}\;v\in {\tilde{V}}_{1},\\ \vdots & \vdots \\ i_{JINF,max,s}, & \text{if}\;v\in {\tilde{V}}_{s}, \end{array}$$ (22)

with *i*_*JINF*,*max*,*j*_ > 0 for all *j* = 0, …, *s*. In order to maintain the correlations between *i*_*JINF*_,_*max*_(*v*), *i*_*IP*3_,_*max*_(*v*) and *t*_1_(*v*), we define similarly *i*_*IP*3_,_*max*_(*v*) = *i*_*IP*3,*max*,*j*_ and *t*1(*v*) = *t*_1,*j*_ for $v\in \tilde{V_{j}}$, *j* = 0, …, *s* with *i*_*IP*3,*max*,*j*_ > 0, *t*_1,*j*_ > 0, so that the rank statistics of ${\left(i_{JINF,max,j}\right)}_{j=0,\ldots ,s}$ corresponds to the rank statistics of ${\left(i_{IP3,max,j}\right)}_{j=0,\ldots ,s}$ and ${\left(1/t_{1,j}\right)}_{j=0,\ldots ,s}$. These models enable us to examine particular features of intercellular Ca^2+^ wave propagation.

#### Model G_R_: Random model

This model aims at considering the natural stochastic properties observed in living systems. Instead of fixing particular values for *i*_*JINF*,*max*_(*v*), *i*_*IP*3,*max*_(*v*) and *t*_1_(*v*), we randomize them with a white noise (identical and independent randomizations), so that

$$i_{IP3,max}\left(v\right)=\mu_{i_{IP3,max}}\;+\;\varepsilon_{v},\;\;\;\forall \;v\in V,$$ (23)

where $\mu_{i_{IP3,max}}$ is the mean of the random variables *i*_*IP*3,*max*_(*v*) and where (*ε*_*v*_) are independent and identically distributed (i.i.d.) normal centered random variables with standard deviation $\sigma_{i_{IP3,max}}$, i.e. $i_{IP3,max}\left(v\right)\;\sim \;N\left(\mu_{i_{IP3,max}},\sigma_{i_{IP3,max}}\right),\;\;\forall \;v\in V$. In order to maintain the correlation between *i*_*JINF*,*max*_(*v*), *i*_*IP*3,*max*_(*v*) and *t*_1_(*v*), the parameters *t*_1_(*v*) and *i*_*JINF*,*max*_(*v*) were randomized as follows. Let (*ψ*_*v*_) be a centered white noise with standard deviation $\sigma_{t_{1}}$ and (*φ*_*v*_) a centered white noise with standard deviation $\sigma_{i_{JINF,max}}$. Let

$$\varepsilon_{\left[1\right]}\leq \varepsilon_{\left[2\right]}\leq \ldots \leq \varepsilon_{\left[nm\right]},\;\;\;\varphi_{\left[1\right]}\leq \varphi_{\left[2\right]}\leq \ldots \leq \varphi_{\left[nm\right]}\;\;\;\;\text{and}\;\;\;\;\psi^{\left[1\right]}\geq \psi^{\left[2\right]}\geq \ldots \geq \psi^{\left[nm\right]}$$

denote the order statistics of the noises (*ε*_*v*_), (*φ*_*v*_) and (*ψ*_*v*_), such that *ε*_[1]_ is the minimum of (*ε*_*v*_) and *ψ*^[1]^ the maximum of (*ψ*_*v*_). For a given node *v* ∈ *V*, if *ε*_*v*_ = *ε*_[*i*]_, then set *ψ*_*v*_ = *ψ*^[*i*]^ and *φ*_*v*_ = *φ*_[*i*]_. The resulting noises (*ψ*_*v*_) and (*φ*_*v*_) are white, but (*φ*_*v*_) is negatively correlated with (*ε*_*v*_) and (*ψ*_*v*_) is positively correlated with (*ε*_*v*_). Finally, set

$$t_{1}\left(v\right)=\mu_{t_{1}}+\;\psi_{v},$$ (24)

$$i_{JINF,max}\left(v\right)=\mu_{i_{JINF,max}}+\;w_{v},$$ (25)

where $\mu_{t_{1}}$ and $\mu_{i_{JINF,max}}$ are the means of the random variables *t*_1_(*v*) and *i*_*JINF*,*max*_(*v*) for all *v* ∈ *V*. With this construction, large values of *i*_*IP3*,*max*_ correspond to small values of *t*_1_. Indeed, the largest *i*_*IP3*,*max*_(*v*) has the smallest value *t*_1_(*v*), the second largest *i*_*IP3*,*max*_(*v*) has second the smallest *t*_1_(*v*), etc.

#### Model G_R,C_: Random model with one particular central zone

Similarly as in the model *G*_*R*_, we define $i_{IP3,max}\left(v\right)\;\sim \;N\left(\mu_{i_{IP3,max,j}},\sigma_{i_{IP3,max,j}}\right)$ for all *v* ∈ *V* and where *j* ∈ {0,1}. Define a small zone of size ${\tilde{n}}_{1}\times {\tilde{m}}_{1}$ at the center of the graph and denote its nodes by ${\tilde{V}}_{1}$. Then, for all $v\in {\tilde{V}}_{1}$, j = 1 and for all other *v*, j = 0. The other parameters are defined analogously, with the correlation assumptions fulfilled as in the random model *G*_*R*_.

All analyses were performed in MATLAB version 2013b (Mathworks Inc., Natick, MA, USA) and the code for the scripts is available in the Supplementary Material (S1 Code).

## Results

### Oscillatory regimes depending on the sensitivity of individual cell in model *G*_*O*_

**Fig 2:**
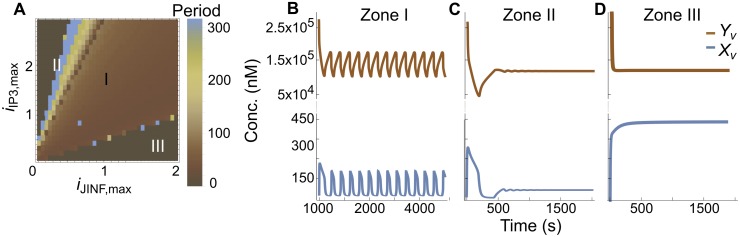
Behavior of a single cell in absence of gap junctional coupling (*d* = 0). (A) The oscillation period is computed for couples (*i*_*JINF*,*max*_, *i*_*IP3*,*max*_). Three zones emerge. Zone I; The Ca^2+^ concentration c_cyt_ oscillates. Zone II (the boundary of zone I); small oscillations occur, but disappear rapidly. Zone III; A rapid saturation in c_cyt_ occurs preventing any oscillations. (B-D) Evolution of c_ER_ (orange traces) and c_cyt_ (blue traces) for parameters chosen in zones I, II and III, respectively.

An isolated cell (model *G*_0_) oscillates in response to a given input encapsulated into the functions *J*_*INF*_ and *InsP*_*3*_ for well-chosen parameters (see [16]). Unless specified, all parameters are set according to Table 1 and the ranges of parameters leading to oscillations are depicted in Fig 2. The period of oscillations (inverse of the frequency) is determined by *i*_*JINF*,*max*_ and *i*_*IP3*,*max*_; three zones can be distinguished. In zone I, the Ca^2+^ concentration oscillates with a constant frequency as exemplified in Fig 2B. In zone II (the boundary of zone I) any small oscillation rapidly vanishes after the initiating stimulation signal (Fig 2C). Finally, in zone III, no oscillations occur and Ca^2+^ concentrations saturate (Fig 2D). The model for each cell has a Hopf bifurcation that separates region III from I and a homoclinic one that separates region I from region II. The system oscillates in a certain range of *i*_*JINF*,*max*_ and *i*_*IP3*,*max*_ values. We thus differentiate two types of cells: self-oscillating ones and non-self-oscillating ones.

### Effect of the gap junctional coupling (*d*) on Ca^2+^ waves in the noise-free models *G*_*D*_

**Fig 3:**
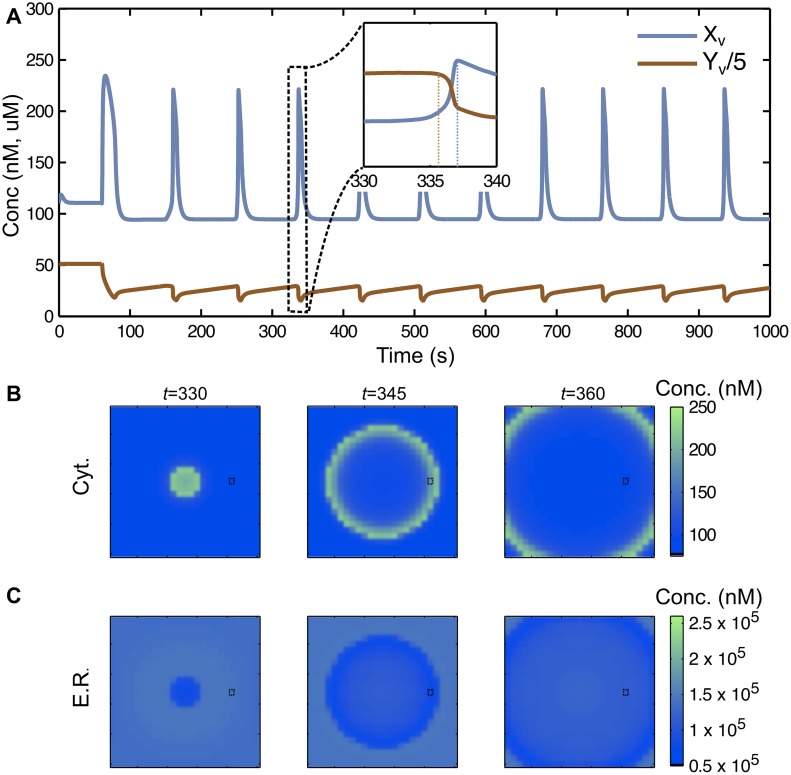
A Ca^2+^ wave emerges from the zone with highest sensitivity. (A) Fluctuations in c_cyt_ (blue) and c_ER_ (orange) of one particular cell. (B) Evolution of the wave between time *t* = 330 and *t* = 360. The panel (A) corresponds to the oscillations of a particular cell (black square). (C) Evolution in the ER component. The central region has parameters set to *i*_*JINF*,*max*,1_ = 1.08, *i*_*IP3*,*max*,1_ = 2.16 and *t*_1,1_ = 72. The gap junctional coupling is moderate/low (*d* = 0.003).

**Fig 4:**
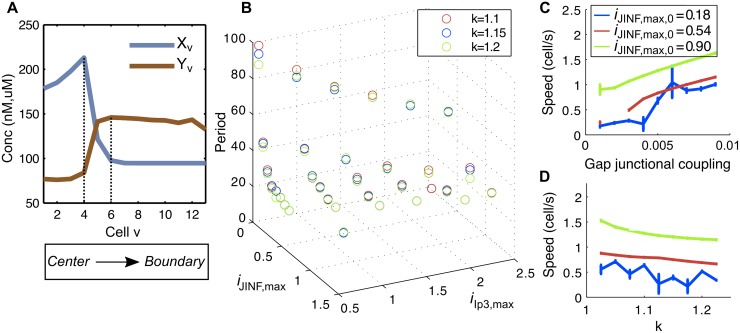
The wave front is initiated in the ER and its speed is determined by the gap junctional coupling *d*. (A) Time is fixed (t = 335) and we represent the wave from the center to the boundary of the graph. Blue and orange traces, represent c_cyt_ and c_ER_, respectively. (B) The parameters are set such that $\frac{i_{JINF,max,1}}{i_{JINF,max,0}}=\frac{i_{IP3,max,1}}{i_{IP3,max,0}}=\frac{t_{1,0}}{t_{1,1}}=k$. The oscillation period decreases with large values of *i*_*JINF*,*max*,0_ and *i*_*IP3*,*max*,0_. (C-D) The speed of the wave is estimated from simulations by computing the time needed for a circular wave to reach the boundary of the graph, when starting from the center. Error bars represent 95% confidence intervals of the mean. In (C) a positive correlation with the gap junctional coupling is found for any value of *i*_*JINF*,*max*,0_. We fix *i*_*JINF*,*max*,0_ = 1.8. In (D), only high values of *i*_*JINF*,*max*,0_ lead to a negative contribution of the parameter *k* to the speed. We fix *d* = 0.003.

Consider a noise-free graph (model *G*_*D*_) with gap junctional coupling *d* > 0 and one particular region at the center of the homogenous, fully dense graph $G_{0}$, where the sensitivity is increased (*i*_*JINF*,*max*,1_ >*i*_*JINF*,*max*,0_, *i*_*IP3*,*max*,1_ > *i*_*IP3*,*max*,0_ and *t*_1,1_ < *t*_1,0_). In this setting, a Ca^2+^ wave emerges from the center of the graph, indicating that the most sensitive cells are the wave initiators (Fig 3 and S1 Movie). The wave front is initiated in the ER as illustrated in Fig 4A. Hence, intercellular Ca^2+^ waves are driven by ER Ca^2+^ release as reported in [29]. This is also in accordance with the findings in [16], where in single cells, oscillations are initiated from within the ER. With a strictly positive gap junctional coupling, single oscillations thus propagate through all connected cells with a velocity controlled by the strength of the gap junctional coupling *d*, as illustrated in Fig 4C. In Fig 4B, we report that *i*_*JINF*,*max*,0_ and *i*_*IP3*,*max*,0_ additionally contribute to the wave speed. Large values of the parameter *d* diminish the period of the wave and thus increase the oscillation frequency. Note however that if parameters *i*_*JINF*,*max*,0_ and *i*_*IP3*,*max*,0_ are too large, the network homogenizes rapidly, so that c_cyt_ reaches saturating values in every cell, thus dampening any oscillatory behavior.

Indeed, parameters *i*_*JINF*,*max*,0_ and *i*_*IP3*,*max*,0_ negatively correlate with the period of oscillation in all cells of the graph as illustrated in Fig 4B. In this figure, the period of oscillation has been calculated for various parameter values of *i*_*JINF*,*max*,0_, *i*_*IP3*,*max*,0_ and a moderate *d*. Both parameters participate to the homogenization of the patterning through the graph. If both parameters *i*_*JINF*,*max*,0_ and *i*_*IP3*,*max*,0_ are sufficiently large, no oscillation is observed. All cells fill up with Ca^2+^ and remain on a stationary state. In Fig 4, we chose $\frac{i_{JINF,max,1}}{i_{JINF,max,0}}=\frac{i_{IP3,max,1}}{i_{IP3,max,0}}=\frac{t_{1,0}}{t_{1,1}}=k>0$, which is seen as an added percentage of sensitivity in the sub-graph $\tilde{G}$. This parameter *k* has no remarkable global effect except that for sufficiently large values of *i*_*JINF*,*max*,0_, it slightly negatively contributes to the wave speed (Fig 4D). In the S2 Text, an extension of this model, which explicitly takes into account the presence of calretinin, shows that this specific buffer has an effects on wave propagation: an increase in calretinin tends to increase the oscillation frequency and the wave speed, as illustrated in Fig. A in S2 Text.

**Fig 5:**
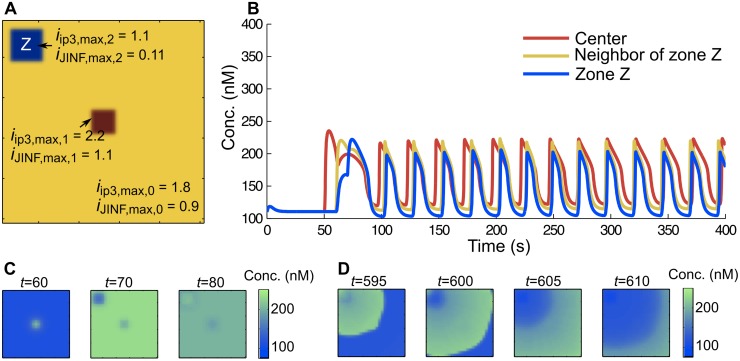
Synchronization of non-self-oscillating cells with their oscillating neighbors. (A) The graph was split into three zones. The central zone is highly sensitive (red). Zone *Z* (blue) is non-sensitive and cells from this zone would not oscillate, if isolated from the others. The rest of the graph (yellow) is set with standard parameters. (B) Oscillations in the center (red), a neighbor of the zone *Z* (yellow) and the zone *Z* (blue). Synchronization occurs rapidly. (C) The wave is initiated in the central zone, propagates through the graph and finally enters zone *Z*. (D) After a long period, the wave starts from neighboring cells of *Z*. This is illustrated in (B), where the yellow and blue peaks follow the red one after a sufficiently long time period (right shift of the start of oscillatory activity).

Interestingly, the synchronization of neighboring cells occurs even if the network contains non-self-oscillating cells. To verify this, we add to the previous network a single region *Z* of cells with parameters (*i*_*JINF*,*max*,2_, *i*_*IP3*,*max*,2_) outside of the range that would allow them to oscillate independently (zone III in Fig 2D). Any oscillations in those cells would rapidly die out (Fig 2C). But since they are coupled to neighboring oscillating cells, they synchronize and oscillate in response to the behavior of their neighbors (Fig 5 and S2 Movie). Ca^2+^ ions transported to non-self-oscillating cells thus evoke changes in their oscillations-properties. This phenomenon is accelerated by higher gap junctional coupling values. In such a configuration, we observe that the wave initiators are shifted from the center to cells adjacent to the region *Z*.

### Effect of randomization and holes in the random model *G*_*R*_

**Fig 6:**
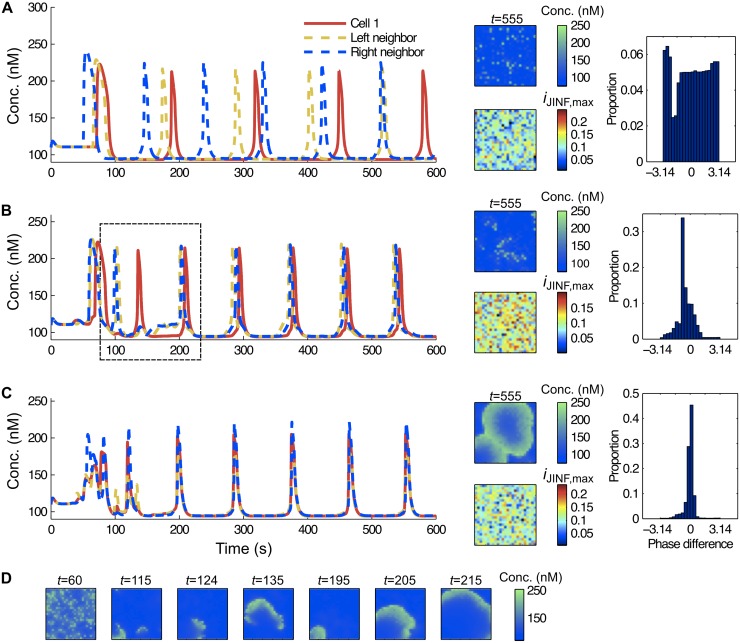
Moderate coupling produces bursting phenomena in noisy systems (model *G*_*R*_ with *p* = 0). (A) Evolution of c_cyt_ in a particular cell of the graph and in two neighbors with no gap junctional coupling (*d* = 0). Changes in c_cyt_ occur independently in each cell and even after long periods (*t* = 555), no coherent (synchronous) behavior is observed (see also S3 Movie). (B) When gap junctional coupling is augmented to low values *d* = 0.0015), the system hardly synchronizes. Small bursting phenomena (between time 100 and 200) are observed before synchronization, see S4 Movie. (C) The gap junctional coupling is set to a high value (*d* = 0.009); synchronization occurs easily between the neighboring cells and a coherent behavior is observed, see S5 Movie. (D) The wave is represented at different time points with the settings of panel (C). Histograms in (A-C) represents the distribution of the phase differences of two arbitrary chosen neighboring cells, illustrating the synchronization measure. Here *m*_*sync*_ = 0.07 in (A), *m*_*sync*_ = 0.19 in (B) and *m*_*sync*_ = 0.58 in (C). All parameters are set according to Table 1 with high noise and $\mu_{i_{JINF,max}}=0.108$.

When considering noise in the system, the same local effects depicted previously (Figs 4 and 5) occur. Neighboring cells, although having different sensitivities, try to synchronize their operation. This is illustrated in Fig 6 and S3–S5 Movies, where we used the random model *G*_*R*_ described in the previous section for a system with strong noise and with *p* = 0, i.e. no links were removed (graph $G_{0}$). The extreme case of no coupling (*d* = 0) leads to independent Ca^2+^ oscillations in each cell (Fig 6A and S3 Movie). For low and moderate couplings, small bursting phenomena are visible before synchronization of two neighboring cells (Fig 6B and S4 Movie). By “burst” we mean that a cell changes its phase during the development of a Ca^2+^ spike resulting in a prolonged irregular Ca^2+^ spike. Moreover, no coherent behavior or typical wave patterns emerge from such situations. Incorporating calretinin into the model has the very interesting effect of promoting the formation of coherent wave patterns. This phenomenon is exemplified in S18 Movie, where the same framework as in Fig 6B is used, with the addition of calretinin (see S2 Text). More generally in Fig. B in S2 Text, we illustrate that a deficiency in coupling can be generally compensated by sufficiently high levels of calretinin from the point of view of synchronization. Notice that strong coupling leads to well-synchronized oscillations, even in the presence of strong noise (Fig 6C and S5 Movie). We observed that when phase synchronization occurs, the initial differences in the amplitudes of individual cells decrease, i.e. the harmonization of the network is also visible as a harmonization of the amplitudes. Highly sensitive cells still initiate Ca^2+^ waves travelling through the network. In such noisy systems, waves can arise from different random places. When they collide, they aggregate or split depending on their velocity and on local properties of the graph.

In the S1 Text, we also consider the effect of InsP_3_ coupling within this random model. In these particular settings, Fig. A in S1 Text illustrates how InsP_3_ and Ca^2+^ oscillations synchronize in an arbitrary cell. S14–S16 Movies show the evolution of Ca^2+^ concentrations when (i) Ca^2+^ coupling is active, but there is no InsP_3_ coupling (S14 Movie), (ii) InsP_3_ coupling is active but there is no Ca^2+^ coupling (S15 Movie) and (iii) Both couplings are active (S16 Movie). There are two important phases in order to compare the three situations. In the beginning, around *t*_1_(*v*), InsP_3_ coupling is very efficiently involved in the synchronization process, but afterwards acts poorly as synchronizing agent (see Fig. B in S1 Text). The reverse is true for Ca^2+^ coupling (Fig. B in S1 Text). All results are reported in Table B in S1 Text.

**Fig 7:**
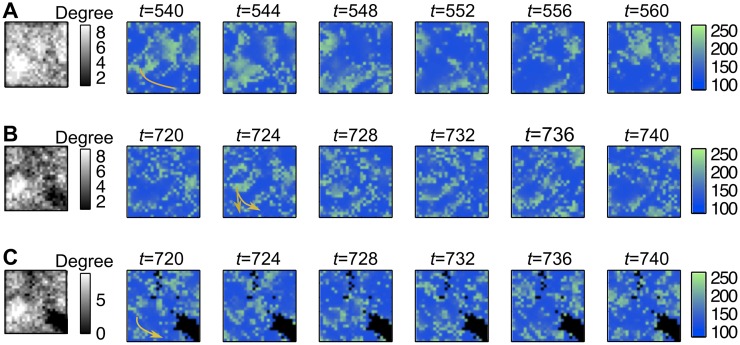
Waves hardly ever propagate through poor linked zones of the graph (model *G*_*R*_ with *p* > 0). (A) Highly linked graph (25% of the links are removed, using *p* = 0.5). The degrees matrix of the graph is presented on the black-white image on the left. Several waves are travelling through the graph. They are considerably slowed down by the low-linked zone (black region) and directed towards high-linked regions (yellow arrow). (B) In a poor-linked graph (40% of the links are removed, using *p* = 1), the same phenomenon is accentuated. (C) Holes are added to the previous graph by removing each node *v* with *F*(*v*) smaller than the 10%-quantile of *F*. This changes completely the behavior of the Ca^2+^ wave propagation. Coupling (*d* = 0.0045) and noise are moderate.

Next we examined the effect of link removal on Ca^2+^ wave propagation i.e. the heterogeneous sparse graph was used (G). Besides the gap junctional coupling (*d*), the density of the connection *p*(*v*) influences the wave propagation. Remember, the parameter *p*(*v*) indicates, whether two neighboring cells are connected or not, while the parameter *d* represents the strength of the coupling if such a connection exists. Hence it is not surprising that increasing the link removal probability *p*(*v*) has a similar effect on the wave propagation as decreasing the strength of the gap junctional coupling *d*. Highly connected cells (regions) enable waves to spread easily, while poorly linked regions will act as a barrier deviating the wave front to another direction. In the extreme case, holes were added to the network (Fig 7 and S6–S8 Movies).

### Spiral waves

The most interesting feature occurring in random systems is the waves-patterning transitions. As explained above, waves initially emerge from highly sensitive cells and spread radially away from them. Such travelling waves commonly appear in many oscillating systems [45]. In inhomogeneous systems built with our model, such patterns do not necessarily stabilize with time. Circle centers move, some waves die out as other gain in strength, collide and even spiraling phenomena are observed (S8 Movie). In this section the different frameworks resulting to spiraling phenomena are explored.

**Fig 8:**
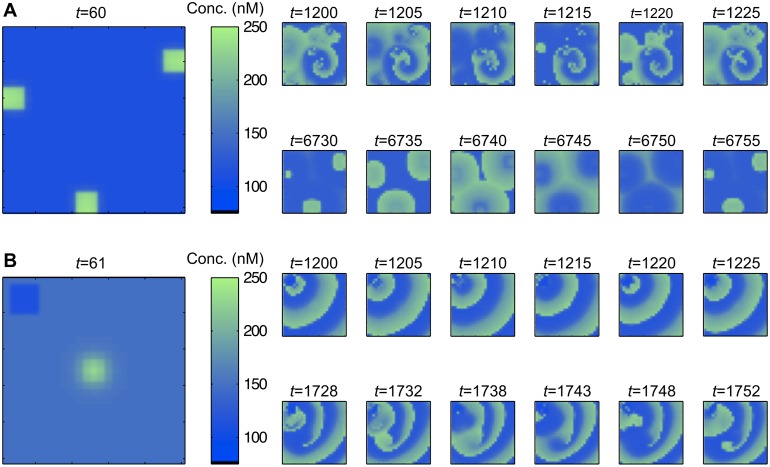
Inhomogeneity in the network and low/moderate gap junctional coupling evoke Ca^2+^ spirals. The model *G*_*D*_ is used. (A) Three regions with higher sensitivity are added on $G_{0}$, with parameters *i*_*JINF*,*max*,*j*_ = 1.08, *i*_*IP3*,*max*,*j*_ = 2.16 and *t*_1,*j*_ = 50, for *j* = 1, 2, 3, creating inhomogeneity (visible at time *t* = 60). Evolution of c_cyt_ at different time points. Spirals form and then break to stable circles centered around the sensitive zones. (B) The framework of Fig 5, with one more sensitive zone at the center and a non-oscillating region in the upper-left corner of the graph enables spiral formation (parameters of Fig 5).

In our settings, surprisingly, even homogenous noise-free networks can exhibit transitions from circles to spiral waves. The cause of this has to be looked for in the inhomogeneity of cell sensitivities. Indeed, if we consider the same framework as in [46], by considering model *G*_*D*_, with three additional small regions where cells are more sensitive, spiral waves appear (see Fig 8A and S9 Movie). These spirals are of transitory nature and the system finally ends up with stable circular waves emanating from the three sensitive zones.

Notice that defining two particular zones is sufficient to create spirals as demonstrated in Fig 8B and S2 Movie. In this figure, the central region is very sensitive and a second area (upper-left) is low sensitive (with parameters in zone III of Fig 2A). After a sufficiently long time, a spiral develops around this second area and moves along the network. This sheds light on the effect of gap junctional coupling. Here its value (d = 0.0045) is moderate. An identical framework, but with a high gap junctional coupling, would prevent spiraling behaviors. Considering two highly sensitive areas leads to the same conclusion, as illustrated in S10 Movie.

Beyond collisions of different travelling circular waves and topological considerations, gap junctional coupling and inhomogeneous activation (as defined by e.g. noise strength) play key roles in spiral generation. Independently of its strength, noise acts as a catalyst for producing spirals. For a low level of noise, moderate gap junctional coupling enables the appearance of transitions between circles and spirals. In highly disordered systems, the level of gap junctional coupling needed to smooth the spirals and obtain stable circles increases. Indeed, a high gap junctional coupling allows waves to spread rapidly among the cells and thus enables a fast synchronization of their behavior (Fig 6C). As such, any inhomogeneity that can be generated from different traveling waves breaks up.

**Fig 9:**
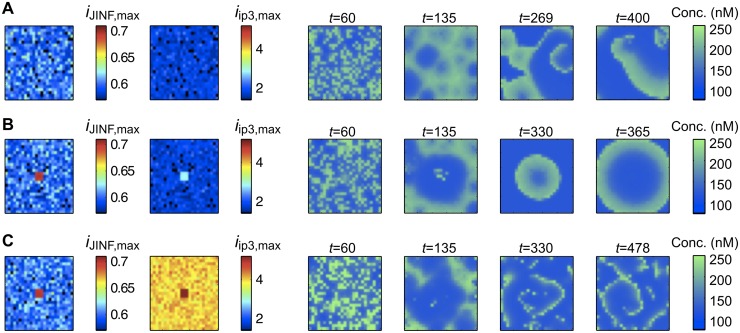
Effect of overstimulation of one parameter in the random models *G*_*R*_ and *G*_*R*,*C*_. The diffusion is moderate and the noise is low. Every panel represents the particular realizations of *i*_*JINF*,*max*_, *i*_*ip3*,*max*_ and c_cyt_ in the graph $G_{0}$ at four particular time points. (A) Model *G*_*R*_. The system is sufficiently sensitive and inhomogeneous to produce spirals. We set $\mu_{i_{IP3,max}}=1.8$ and $\mu_{i_{JINF,max}}=0.6$. (B) A more sensitive zone is added at the center of the graph (model *G*_*R*,*C*_) with $\mu_{i_{IP3,max,1}}=2.6$ and $\mu_{i_{JINF,max,1}}=0.7$ (again with $\mu_{i_{IP3,max,0}}=1.8$ and $\mu_{i_{JINF,max,0}}=0.6$). Concentric circles appear. (C) The previous system is overstimulated with $\mu_{i_{IP3,max,1}}=5.5$ and $\mu_{i_{IP3,max,0}}=4$ (other parameters as in (B)), which results in incoherent behavior and spirals. All parameters are set according to Table 1, except if specified. The standard deviations in the central zone are $\sigma_{i_{IP3,max,1}}=0.01$, $\sigma_{i_{JINF,max,1}}=0.001$ and $\sigma_{t_{1},1}=0$. $\mu_{t_{1},1}$ is set to the minimal value of *t*_1_(*v*)for $v\in V_{0}\backslash {\tilde{V}}_{1}$.

To explore the particular effect of inhomogeneous activation, the noise is set to a low value. In Fig 9A (and S11 Movie) the random model *G*_*R*_ is used with a relatively high sensitivity. Due to the noise, the system is inhomogeneous enough to produce spirals, as locally explained in Fig 8A–8B. Turning to model *G*_*R*,*C*_, by adding a small central zone with higher sensitivity regulates the behavior of the system and concentric circles develop (Fig 9B and S12 Movie). In this case the sensitive center enables the homogenization of the whole network. This occurs in a similar way as what is shown in the noise-free framework of Fig 3. Increasing simultaneously the two main frequency-determining parameters, $\mu_{i_{IP3,max}}$ and $\mu_{i_{JINF,max}}$, results in similar concentric circles, but with higher frequencies (see S19 Movie, where the same parameters as in Fig 9C are used with $\mu_{i_{JINF,max,0}}=0.9$ and $\mu_{i_{JINF,max,1}}=1$). However, very high values for one parameter (e.g. $\mu_{i_{IP3,max}}$ as in Fig 9C and S13 Movie) shifts the model to non-organized wave propagation and creates spirals again; the sensitive center is no more sufficient to homogenize the whole network. Our explanation for this phenomenon is the following: increasing one parameter excessively increases the number of non-self-oscillating cells (Fig 2A Zone III), but when increasing simultaneously both parameters, most cells remain in the oscillating Zone I and the network is able to produce rhythmic concentric circles The effect of specific buffers (calretinin) on $\mu_{i_{IP3,max}}-$ overstimulated systems is investigated in S2 Text. Our general finding is that Ca^2+^ buffers promote coherent behavior in these situations.

## Discussion

Over the last 25 years, many different models have been developed to describe Ca^2+^ oscillations in cells [23,24,47]; new methods including high-resolution spatiotemporal recordings of Ca^2+^ signals enabled the reconsideration and further development of the different models. As an example, the simultaneous monitoring of c_cyt_ and InsP_3_ production has revealed that Ca^2+^ oscillations are not the direct consequence of fluctuations in [InsP_3_] [29]. Simultaneous monitoring of c_cyt_ and c_ER_ showed a time shift between the maximum of c_cyt_ and the minimum in c_ER_ during a Ca^2+^ spike [48–51], findings that have allowed for a better understanding of the mechanisms implicated in oscillations. The continuous loading of the ER with Ca^2+^ during the interspike periods followed by a rapid release during the Ca^2+^ spike in c_cyt_ results in sawtooth-like Ca^2+^ oscillations in c_ER_ [16]. This indicates that the loading state of the ER is an essential parameter to understand Ca^2+^ oscillations in the cytosol. A next Ca^2+^ spike can be generated only, if c_ER_ reaches a certain threshold value [52]. This threshold is determined by the prevailing InsP_3_ concentration [42]. The replenishment of the ER Ca^2+^ store is modulated by a constant Ca^2+^ influx across the plasma membrane. An increase in the Ca^2+^ influx rate leads to a higher frequency of Ca^2+^ oscillations, while decreasing the Ca^2+^ influx reduces the frequency [16,26,53]. In some conditions, mitochondrial Ca^2+^ transport (uptake and release) was found to substitute for the plasmalemmal Ca^2+^ exchange function, thus rendering the oscillations independent of extracellular Ca^2+^ [26]. The magnitude of the Ca^2+^ transport into mitochondria was also found to influence the Ca^2+^oscillation frequencies [54]. Often it is the refilling of the ER that sets the oscillation period (frequency), not the InsP_3_R dynamics. The model proposed in our previous works [16,26] has demonstrated to be coherent with the above-mentioned experimental findings at the single-cell level (zero dimension). In this study, we showed that this single-cell model in a 2D framework is a useful tool for the prediction and understanding of several phenomena in naturally-occurring multicellular noisy systems. Among existing models for intercellular Ca^2+^ waves, the key limitations are the size of the system (two [27] or three cells [55]), restriction of cell activation to a single cell [56] or the fact that cells with altered sensitivities are restricted to few areas in an otherwise noise-free system [46]. To the authors' best knowledge, the model that we propose in the present study is the first to address simultaneously those limitations.

Our model revealed the ER to be the initiation site of the Ca^2+^ phase wave front (Figs 3A and 4A). Thus, intercellular Ca^2+^ phase waves are driven by an initiative wave front starting in the ER; even if the neighboring cells might communicate with each other by exchanging their cytosolic but not luminal Ca^2+^ ions via gap junctions. This model prediction has already been proven experimentally. Keller and coworkers have found in guinea heart myocytes that Ca^2+^ waves in c_cyt_ are driven by “sensitization” wave fronts in c_ER_ [57]. Differently from Ca^2+^ phase waves, the initial wave front of Ca^2+^ trigger waves starts from within the cytosol. Another difference between the two types of waves is that two Ca^2+^ phase waves annihilate each other when they collide, while two Ca^2+^ trigger waves add up to generate a new wave of greater amplitude.

It has been known for a long time that gap junctions are permeable both to InsP_3_ and Ca^2+^ ions [58]. However, because Ca^2+^ but not InsP_3_ is strongly buffered by cytoplasmic proteins and/or unidentified immobile buffers, Ca^2+^ movement within a cell is very restricted and slower than that of InsP_3_ [59]. Thus InsP_3_ is more likely to diffuse to greater distances and subsequently to mediate intercellular Ca^2+^ waves [21]. Although this concept is quite attractive, one has to take into account that (i) Albritton et al. [59] measured the Ca^2+^ diffusion in cell extracts, where the Ca^2+^ pumps and InsP_3_-metabolizing enzymes were blocked, i.e. not in physiological conditions. (ii) To generate a long distance Ca^2+^ wave, Ca^2+^ ions do not need to travel for long distances; it is enough to diffuse to the next InsP_3_R or RyR. More precisely, since these receptors are organized in clusters, Ca^2+^ ions only have to diffuse from one cluster to the neighboring cluster. Since the ER consists of a network in the cytoplasm filling almost the entire cell, the cluster-to-cluster distances between adjacent cells should be of similar magnitude than that of intracellular cluster-to-cluster distances, i.e. based on the images presented in Chalmers et al. [60], approximately 1 μm. (iii) Ca^2+^ buffer proteins not only take up Ca^2+^ ions, but to the same extent, also release Ca^2+^ ions. Depending on their Ca^2+^-binding parameters (fast or slow kinetics) they can promote or inhibit intracellular Ca^2+^ wave formation [61]. Most probably, they have similar effects on intercellular wave formation, yet no experimental data are available. As a preliminary result in the S2 Text, we have already simulated the effect of a specific Ca^2+^ buffer, calretinin, on wave propagation. Our model predicts that calretinin promotes intercellular synchronization. (iv) As presented in the introduction, there are two types of Ca^2+^ waves: Ca^2+^ trigger waves and Ca^2+^ phase waves. If we complete our model with the gap junctional transport of InsP_3,_ this results in Ca^2+^ trigger waves, since only few cells get activated before activation of the large majority of the other cells. In this case the gap junctional transport of InsP_3_ is the main agent harmonizing the initial Ca^2+^ signal, in agreement with previous experimental results [21]. However, the existence of individual Ca^2+^ oscillatory machinery and the individual stimulation of each cell, as in our model, allows the generation of phase waves in which the emergence of the waveform is due to Ca^2+^ release from adjacent oscillating cells slightly differing in their oscillation phase.

In summary, our model shows that both Ca^2+^ ions and InsP_3_ are likely the synchronizing agents, possibly in a synergistic way. InsP_3_ is more involved in the formation of the initial Ca^2+^ trigger waves, while Ca^2+^ ions serving as a coupling agent are more implicated in the later ones indicating that Ca^2+^ phase waves are dominant at the later stages of a model experiment (See S1 Text). Of note, in other studies, the authors have concluded that only InsP_3_ may serve as the coupling agent [55,62], reporting that the pertaining actual [InsP_3_] is the only frequency-determining factor. In our model, we identified two factors determining the Ca^2+^ oscillation frequencies, i.e. *i*_*JINF*,*max*_ and *i*_*IP3*,*max*_.

Stimulation of primary mesothelial cells induces Ca^2+^ responses showing a wide range of different oscillatory patterns within a single, supposedly homogenous cell population. Since a single cell type may exhibit most, if not all, of the different types of oscillatory patterns, most likely each cell contains all components of the Ca^2+^ signaling toolkit (possibly to different extents) required to generate the full range of oscillatory patterns and spreading Ca^2+^ waves [25]. Our model indicates that different spatiotemporal patterns of intercellular Ca^2+^ signals are mostly the consequence of different strengths of coupling via gap junctions. Non-synchronous oscillations in individual cells is favored when gap junctional coupling is weak as was observed in cultured primary mesothelial cells [16]. Moderate levels of gap junctional coupling led to temporally and spatially restricted Ca^2+^ bursts (Fig 6). Nevertheless, other mechanisms can also be involved in the formation of Ca^2+^ bursts [63–65]. This type of oscillations and waves was found in pancreatic beta cells upon glucose stimulation [66]. Strong gap junctional coupling resulted in Ca^2+^ waves, even if individual cells had different sensitivities with respect to stimulation. Synchronous smooth muscle cell-mediated contractions of the uterus driven by Ca^2+^ waves [67,68] are a typical example of strong coupling. The influence of gap junctional coupling on the generation and spreading of intercellular Ca^2+^ waves was experimentally revealed by analysis of GT-1 cells, a cell line derived from immortalized Luteinizing Hormone-Releasing Hormone neurons. GT-1 cells were then further subcloned to result in lines GT1-1, GT1-3 and GT1-7 cell lines differing in expression levels of connexin 26 (Cx26) [69,70]. Low Cx26-expressing GT1-7 cells mostly displayed frequent spontaneous asynchronous Ca^2+^ oscillations, while high Cx26-expressing GT1-1 cells showed spontaneous intercellular Ca^2+^ waves, completely in line with our model. The occurrence of Ca^2+^ waves strongly depends on cell-cell contact probability and moreover the strength of gap junctional coupling. Our model also predicts that in a noisy system, in which each cell has an individual sensitivity to stimulation, the most sensitive cells act as the wave initiator cells.

Physiological cellular responses to evoked Ca^2+^ signaling are cell-type dependent: Ca^2+^ signals elicit contraction in muscle cells [71], neurotransmitter release in neurons [72] and e.g. insulin secretion in pancreatic beta cells [73]. Ca^2+^ signals in immune cells participate in the regulation of cell differentiation, gene transcription and effector functions [74]. They are also involved in the regulation of cell proliferation of cancer cells [75]. Intracellular Ca^2+^ signaling, frequently in the form of Ca^2+^ oscillations, activates specific enzymes and transcription factors in a cell, which are often involved in cell proliferation [76]. Ca^2+^ signals are usually not restricted to individual cancer cells, but are propagated to neighboring cells in the form of intercellular Ca^2+^ waves, usually Ca2+ trigger waves are observed [77]. Assuming that intra- and intercellular Ca^2+^ signaling is the major way by which cells encode and transmit information, it is likely that during the passageway of a Ca^2+^ wave, several Ca^2+^-dependent targets in affected cells would be activated and/or deactivated. Our model predicts that Ca^2+^ waves play an important role in the harmonization of evoked responses i.e. Ca^2+^ wave initiator cells are capable of activating neighboring cells, even when those cells would not oscillate by themselves; however they start to oscillate and hence support waves when coupled to other cells. Thus, a highly sensitive cell may trigger cell division even if the neighboring cells are not sensitive enough to mitogenic stimuli. The exploration of the details on Ca^2+^ wave-dependent harmonization of Ca^2+^-related cellular responses remains an interesting topic to be investigated experimentally in the future.

Our model is capable of producing both circular waves and spirals within a specific range of parameters. Systems producing spirals, such as the famous Fitzhugh-Nagumo equations have been mathematically analyzed [45,78–80] and several ways to create spirals in oscillating systems was proposed in the work of Mckenzie [81]. In biological systems, Ca^2+^ spirals are observed in “overstimulated” conditions. Frog oocyte overexpressing muscarinic acetylcholine receptor produce spiral waves in c_cyt_ upon stimulation [82].

During normal physiological function of many organs, directed rhythmic Ca^2+^ waves are required, i.e. Ca^2+^ phase waves propagating along a certain direction. For instance, rhythmic Ca^2+^ phase waves are required for the contraction of the gastric pylorus [83], uterus [67,68], intestine [84] or urinary bladder [85]. Heart contraction is even more orchestrated with very fast directed Ca^2+^ phase waves, but in this case the gap junctional transmission of action potentials is thought to be the relevant synchronizing process [22]. Nevertheless, it is not excluded that gap junctional transport of Ca^2+^ and InsP_3_ also plays role in the synchronization. This might explain the phenomena of delayed after-depolarization [86] or arrhythmias associated with altered operation of InsP_3_R [87]. In our model, directed waves observed as circular rings can be generated even in noisy systems, if there is one highly sensitive, wave initiator region. Incorporating one highly sensitive region is an effective way to harmonize networks and to determine the wave direction within a broad frequency range. However, the regulated network collapses, if the number of non-oscillating “signal-plateau” cells increase. Cells usually show signal-plateau response at high stimulation intensity, e.g. by strongly elevated levels of InsP_3_ [29]. This corroborates the experimental observations in biological systems, i.e. spirals appear as a consequence of very extensive stimulation in spatially connected systems [82,88].

From a general viewpoint, noise can arise in any other excitable oscillating network, in which the individual units are connected by different synchronizing agents. Examples for coupled excitable units exist in the field of Biology (synchronization of cellular clocks in the suprachiasmatic nucleus [89]), Physics (Josephon junction circuits [90]), Chemistry (e.g. Belousov–Zhabotinsky reaction [91] or catalytic oxidation of carbon monoxide on a platinum surface [92]) or Social Sciences (social interaction such as waving of a human crowd during a football match[93], an audience clapping in synchrony [94], flashing of fireflies [95] or cricket chirps [96]). We propose that phenomena that we observed in our model might be extrapolated to other systems. For example the Kuramoto model [97] predicts a transition with increasing global coupling strength, at which the oscillators with originally different frequencies become coherent. Also, the global coupling strength was investigated in the limit of large number of oscillating sites [98,99]. Critical coupling was found to differentiate coherent and non-coherent macroscopic behaviors. Their general methods allowed to reducing the involved dynamical description of coupled equations to a finite number of differential equations for the macroscopic state of a system. We demonstrated how local coupling is involved as a globally synchronizing agent in our model, quantified by our synchronization index. Locally, this transition would occur around the “highly sensitive region” and this region would be the initiator (pacemaker) of the subsequent phase waves in originally non-synchronized noisy systems. The “highly sensitive region” might represent the area of higher frequencies or the area with a higher density of links depending on the system. Based on our observation that in all cases, a highly sensitive region gradually determined the behavior of the entire system, as is also observed in many model structures (S1–S3 Texts), one can deduce some general conclusions. This phenomenon may occur if I) each unit has its own machinery for the generation of oscillations, II) there is a certain level of noise within the system, III) there is one or more coupling agents modifying the oscillation frequencies of the coupled units and IV) there is a highly sensitive region allowing for a global synchronization.

## Supporting Information

S1 Movie Circular waves in a noise-free model emerge from highly sensitive cells. Simultaneous changes in c_cyt_ (left panel) and c_ER_ (right panel) have been shown. (AVI)

S2 Movie Non-self-oscillating cells synchronize with their oscillating neighbors and produces spiral waves. (AVI)

S3 Movie Independent evolution of the cytoplasmic Ca^2+^ concentrations c_cyt_ without gap junctional coupling (*d* = 0) in the random model. (AVI)

S4 Movie Bursting phenomena with moderate gap junctional coupling in the random model. (AVI)

S5 Movie Rapid synchronization with high gap junctional coupling in the random model and appearance of spiraling phenomena. (AVI)

S6 Movie Wave propagation in the random model in a highly linked graph. (AVI)

S7 Movie Wave propagation in the random model in a poorly linked graph. (AVI)

S8 Movie Wave propagation in the random model with holes. (AVI)

S9 Movie Appearance of spirals in a noise-free model with three highly sensitive zones. (AVI)

S10 Movie Appearance of spirals in a noise-free model with two highly sensitive zones. (AVI)

S11 Movie A sensitive random model with low noise evokes spirals. (AVI)

S12 Movie A sensitive random model with low noise and an additional sensitive central zone produces concentric circular waves. (AVI)

S13 Movie Setting one parameter above the “physiological” limit in the random model with low noise results in non-organized wave propagation and spirals. (AVI)

S14 Movie Wave propagation in the random model with Ca^2+^ coupling and no InsP_3_ coupling. (AVI)

S15 Movie Wave propagation in the random model with InsP_3_ coupling and no Ca^2+^ coupling. (AVI)

S16 Movie Wave propagation in the random model with InsP_3_ coupling and Ca^2+^ coupling. (AVI)

S17 Movie Wave propagation in the deterministic model with CR. (AVI)

S18 Movie Adjunction of CR in the random model helps synchronization. (AVI)

S19 MovieA sensitive random model with low noise and an additional sensitive central zone produces concentric circular waves (higher $\mu_{i_{IP3,max}}$ and $\mu_{i_{JINF,max}}$ values).(AVI)

S1 Code MATLAB Code (ZIP)

S1 Text Complementary model for InsP_3_ coupling. (PDF)

S2 Text Complementary model for a specified buffer, calretinin. (PDF)

S3 Text Robustness of the model. (PDF)
