# Supplementary material for: The Effect of Gap Junctional Coupling on the Spatiotemporal Patterns of Ca2+ Signals and the Harmonization of Ca2+-Related Cellular Responses
Source: PLoS Comput Biol. 2016 Dec 27;12(12):e1005295. doi: 10.1371/journal.pcbi.1005295 (PMC5226819; doi:10.1371/journal.pcbi.1005295)
Supplement: S1 Text — (PDF) [file pcbi.1005295.s021.pdf]

Complementary model for $InsP_3$ coupling

Tanimura et al. [1] demonstrated cell type-dependent differences in $InsP_3$ dynamics: i.) Ca^{2+} spikes are generated in the absence of synchronous $InsP_3$ fluctuations and ii.) Ca^{2+} spikes are connected by $InsP_3$ fluctuations with a small time delay. In the second case, we have to consider additional $InsP_3$ production by c_{cyt}-dependent phospholipase C activation and $InsP_3$ might play a role in the intercellular Ca^{2+} synchronization. Instead of Eq. (20), we consider that the $InsP_3$ concentration, denoted here by $I(v,t)$, satisfies the following differential equation

$$\frac{\partial I(x,v,t)}{\partial t} = I_G(v,t) + I_{Ca}(x) - I_{DEG}(v,t) + d_I \sum_{\{v,u\}\in\mathcal{E}} (I(u,t) - I(v,t)) \quad (1)$$

where I_G denotes the G-protein dependent $InsP_3$ production, I_{Ca} the c_{cyt}-dependent $InsP_3$ production, I_{DEG} the degradation and d_I the gap junctional permeability of $InsP_3$.

The G-protein dependent $InsP_3$ production is related to stimulus intensity and is modeled as

$$I_G(v,t) = \begin{cases} 0.015\,, & \text{if } t < t_1(v) \\ i_{G,max}(v)\frac{t-t_1(v)}{K_G+t-t_1(v)}, & \text{if } t \geq t_1(v) \end{cases} \quad (2)$$

where K_G is a positive constant and, $i_{G,max}(v)$ as well as $t_1(v)$ can take different values among the cells network, enabling the actual $InsP_3$ concentration to be inhomogeneous in space. The degradation rate of $InsP_3$ is given by the following equation:

$$I_{DEG}(v) = r_{u1} \cdot I(v), \quad (3)$$

with r_{u1} a positive constant. Because the activity of c_{cyt}-dependent phospholipases increases suddenly above resting Ca^{2+} values [2], the c_{cyt}-dependent $InsP_3$ production is modeled by the following function of the cytosolic Ca^{2+} concentration x,

$$I_{Ca}(x) = r_{u2} \cdot \frac{x^3}{K_{Ca}^3 + x^3} \quad (4)$$

with r_{u2} and r_{u3} positive constants. All parameter values are presented in Table A. For the simulations, the values of $i_{G,max}(v)$ are assigned in the same manner as $i_{ip3,max}(v)$, see Table 1 in Main Text.

	Parameter name	Value
Constants	K_G	6 s
	r_{u1}	1.05 /s
	r_{u2}	200 nM/s
	K_{Ca}	1000 nM

Table A. Parameters for $InsP_3$ kinetics.

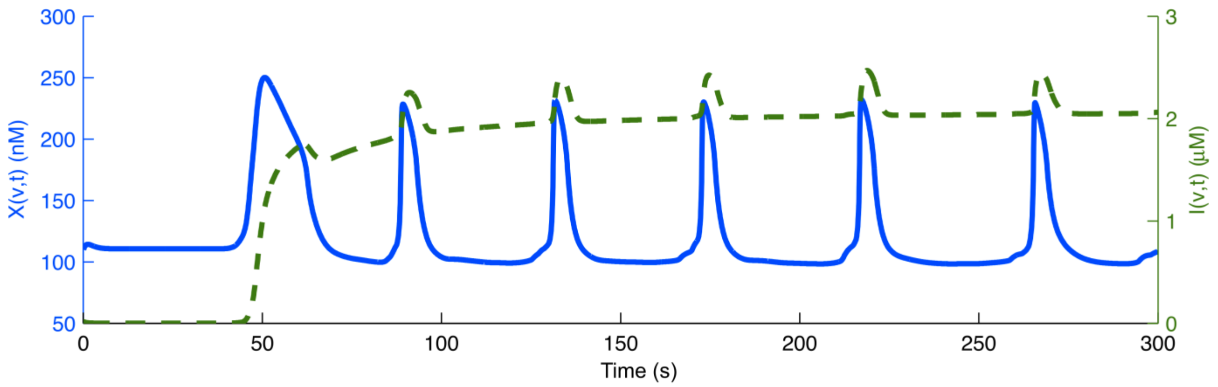

Fig A. Simultaneous changes of c_{cyt} and [$InsP_3$], when considering both $InsP_3$ and Ca^{2+} diffusion The conditions are the ones of model G_R with moderate noise and an identical gap junction coupling parameter for Ca^{2+} and $InsP_3$: $d = d_I = 0.003$. See S16 Movie.

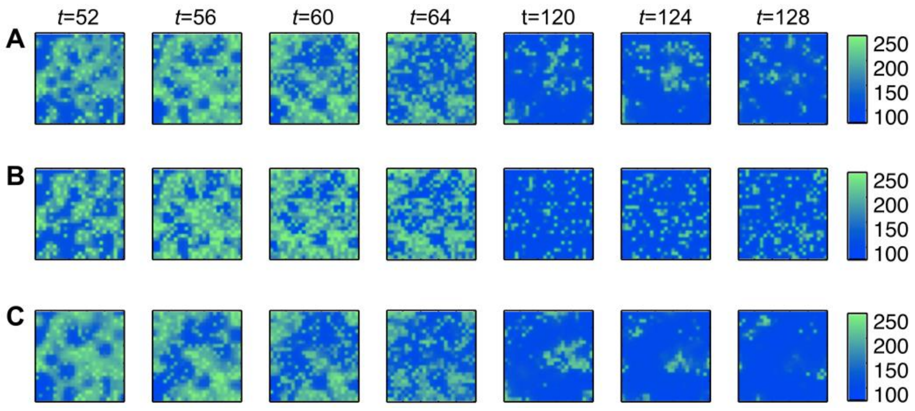

Fig B. Differences in Ca^{2+} propagation for different types of couplings in random model G_R (A) Only Ca^{2+} coupling ($d = 0.003$ and $d_I = 0$, see S14 Movie). (B) Only $InsP_3$ coupling ($d = 0$ and $d_I = 0.003$, see S15 Movie). (C) Ca^{2+} and $InsP_3$ couplings ($d = 0.003$ and $d_I = 0.003$, see S16 Movie). Noise is set to moderate levels (see Table 1 in Main Text). Numerical estimations of

synchronization are reported in Table B of this supplementary text and show that $InsP_3$ coupling is more efficiently involved in synchronization around the first Ca^{2+} peak ($t = 52 - 64$). Afterwards, Ca^{2+} coupling more efficiently synchronizes Ca^{2+} oscillations.

	**35 – 160 (s)**	**161 – 600 (s)**	**35 – 600 (s)**
Only Ca^{2+} coupling	0.118 ± 0.003	0.241 ± 0.004	0.185 ± 0.003
Only $InsP_3$ coupling	0.651 ± 0.015	0.069 ± 0.003	0.103 ± 0.004
Ca^{2+} and $InsP_3$ coupling	0.136 ± 0.003	0.305 ± 0.005	0.246 ± 0.003

Table B. Values of the synchronization index m_{sync} (See Main Text for details) The parameters are the ones used for producing Fig. B of this supplementary text (moderate noise). We observe how $InsP_3$ coupling is involved in synchronization when the signal starts (around $t_1 = 60$, i.e. for $35 < t < 160$). Afterwards, Ca^{2+} coupling is more efficient in synchronizing cell behaviors. See also S14-16 Movies.

References

1. Tanimura A, Morita T, Nezu A, Shitara A, Hashimoto N, et al. (2009) Use of Fluorescence Resonance Energy Transfer-based Biosensors for the Quantitative Analysis of Inositol 1,4,5-Trisphosphate Dynamics in Calcium Oscillations. J Biol Chem 284: 8910-8917.
2. Pawelczyk T, Matecki A (1997) Structural requirements of phospholipase C delta1 for regulation by spermine, sphingosine and sphingomyelin. Eur J Biochem 248: 459-465.
