# Supplementary material for: The Effect of Gap Junctional Coupling on the Spatiotemporal Patterns of Ca2+ Signals and the Harmonization of Ca2+-Related Cellular Responses
Source: PLoS Comput Biol. 2016 Dec 27;12(12):e1005295. doi: 10.1371/journal.pcbi.1005295 (PMC5226819; doi:10.1371/journal.pcbi.1005295)
Supplement: S2 Text — (PDF) [file pcbi.1005295.s022.pdf]

Complementary model for a particular Ca^{2+} buffer, calretinin

In a broad sense, cytosolic Ca^{2+} buffers are all Ca^{2+}-binding proteins. The effect of non-specified Ca^{2+} buffers, including immobile and mobile buffers, is incorporated in the model parameters γ, J_{EFF} and J_{INF}. In a stricter sense, mobile Ca^{2+} buffers comprise specific subsets of Ca^{2+}-binding proteins such as calretinin (CR), calbindin D-28k and parvalbumin. Each Ca^{2+} buffer has different properties and differently modifies the Ca^{2+} signals and they need therefore to be studied separately. CR is of particular interest, since its expression is known to be upregulated during tumorigenic transformation of mesothelial cells and embryogenesis [1,2]. Here, we hence propose an extension of our model, allowing the study of the potential effect of this particular protein on Ca^{2+} wave propagation.

CR has four high-affinity Ca^{2+}-binding sites and one low-affinity binding site. The binding kinetics of each of these sites is modeled with a Hill function of x, the Ca^{2+} concentration in the cytosol, so that the average number of occupied Ca^{2+}-binding sites of CR is

$$\eta(x,t) = 4 \cdot \frac{x^h}{{K_{d1}}^h + x^h} + \frac{x}{K_{d2} + x} \qquad (1)$$

with K_{d1} the dissociation constant for the high-affinity Ca^{2+}-binding sites, $h > 1$ their Hill coefficient (positive cooperativity) and with K_{d2} being the dissociation constant for the low-affinity Ca^{2+}-binding site. The parameter values are taken from the study of Faas et al. [4] and are listed in Table A. The Ca^{2+} flux resulting from binding or releasing Ca^{2+} ions from CR to the cytosol J_{CR} is thus proportional to the temporal changes in the number of Ca^{2+}-occupied sites of CR and the cytosolic concentration of CR, defined by c_{CR}, i.e.

$$J_{CR}(x,t) = c_{CR} \cdot \frac{\eta(x,t-\Delta t) - \eta(x,t)}{\Delta t}, \qquad (1)$$

where Δt is the discretization time in our simulations and is included here for implementation purposes. Due to the relatively low discretization time ($\Delta t = 0.1$ s) of our model we had to neglect the fast kinetics of Ca^{2+} binding to CR which occurs in the millisecond range (fast buffer). Neglecting the fast kinetics of CR limits our model to this relatively slow timescale, and as such, for this model, all parameters and equations are left unchanged, except for Eq. (9),

$$f(x,y,v,t) = -J_{EFF}(x) - J_{SERCA}(x) + J_{EREFF}(x,y,v,t) + J_{ERLEAK} + J_{CR}(x,t) \qquad (3)$$

In our simulations, we varied the parameter c_{CR} to investigate the effect of a specific buffer (CR) present at different intracellular concentrations on the intercellular Ca^{2+} transport and the associated intercellular Ca^{2+} waves.

	Parameter name	Value
Constants	h	1.9
	K_{d1}	1.4 μM
	K_{d2}	36 μM

Table A. Parameters for CR kinetics See Pecze et al. [3] for more details.

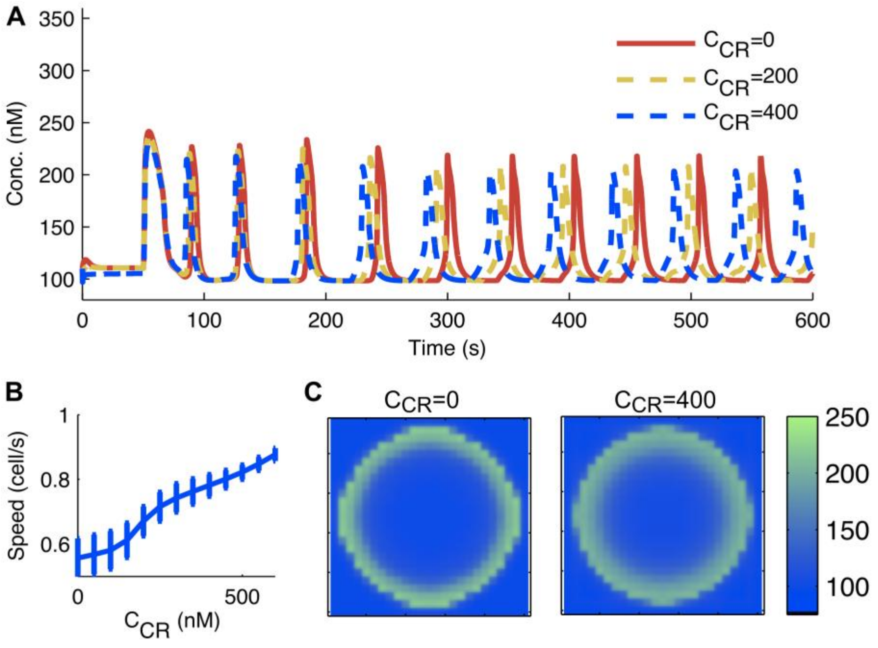

Fig A. Effect of CR on wave properties in the model $\boldsymbol{G_D}$ (A) Ca^{2+} oscillations in the cytosol of one particular cell for different concentrations of CR. High CR concentrations increased the oscillation frequency, reduced the amplitude of Ca^{2+} spikes and increased the spike duration (half-width). (B) The intercellular wave speed is increased with increasing CR concentrations. The estimation was performed as in Fig. 4, Main Text with a 95% confidence interval. The network contains one central zone with higher sensitivity ($i_{JINF,max,0} = 1.8$, $i_{IP3,max,0} = 0.18$ and $i_{JINF,max,1} = 2.16$, $i_{IP3,max,1} = 0.216$) and the Ca^{2+} coupling is set to $d = 0.003$. (C) For all tested CR concentrations, the wave is circular, emerging from the central sensitive zone and travelling towards the boundary (see S17 Movie).

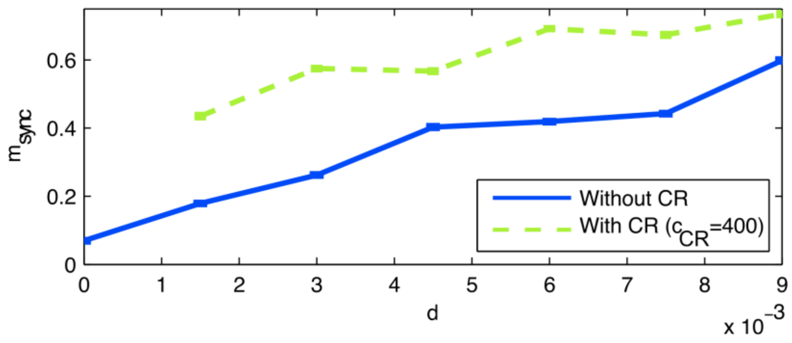

Fig B. CR promotes synchronization and coherent Ca^{2+} patterns in the model $\boldsymbol{G_R}$ A particular network is simulated with similar parameters as in Fig. 6 in Main Text (high noise). Without CR (blue line), stronger couplings (d) lead to more synchronized systems. In the presence of CR, synchrony is increased at all coupling strengths (green dotted line shown for $c_{CR} = 400$). See also the Main Text for the definition of the synchronization parameter m_{sync}.

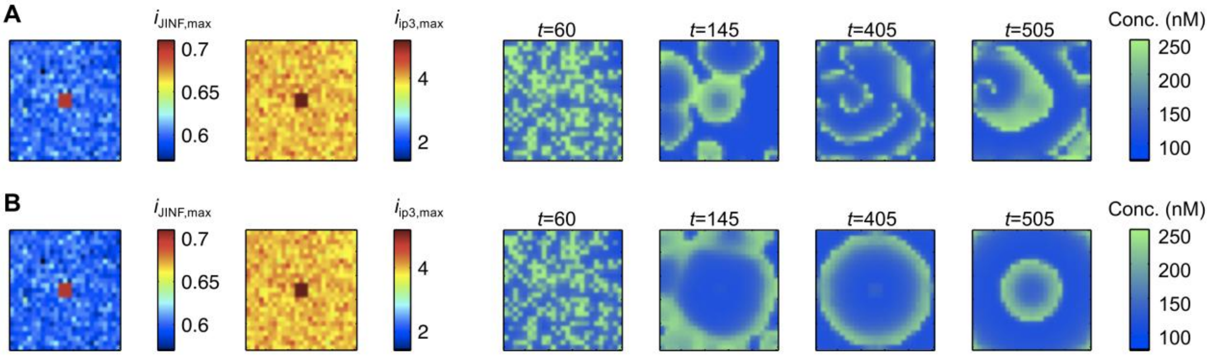

Fig C. CR promotes synchronization in the random overstimulated $\boldsymbol{G_{R,C}}$ model. (A) The same simulation as in the main text (Fig. 9C) with a slightly stronger coupling ($d = 0.006$) is performed. A more sensitive zone is added at the center of the graph (model $G_{R,C}$) with $\mu_{i_{JINF,max,0}} = 0.6$ and $\mu_{i_{JINF,max,1}} = 0.7$ and the system is overstimulated with $\mu_{i_{IP3,max,1}} = 5.5$ and $\mu_{i_{IP3,max,0}} = 4$. This results in spirals. (B) Adding a sufficiently high amount of calretinin ($c_{CR} = 400$ nM) in the situation (A) smoothens the whole behavior and circular waves emerge. All parameters are set according to Table A, unless when specified otherwise. The noise is low and the standard deviations in the central zone are $\sigma_{i_{IP3,max,1}} = 0.01$, $\sigma_{i_{JINF,max,1}} = 0.001$ and $\sigma_{t_1,1} = 0$. $\mu_{t_1,1}$ is set to the minimal value of $t_1(v)$ for $v \in V_0 \backslash \tilde{V}_1$.

References:

1. Gotzos V, Vogt P, Celio MR (1996) The calcium binding protein calretinin is a selective marker for malignant pleural mesotheliomas of the epithelial type. Pathol Res Pract 192: 137-147.
2. Blum W, Pecze L, Felley-Bosco E, Schwaller B (2015) Overexpression or absence of calretinin in mouse primary mesothelial cells inversely affects proliferation and cell migration. Respir Res 16: 153.
3. Pecze L, Blum W, Schwaller B (2015) Routes of Ca2+ Shuttling during Ca2+ Oscillations: FOCUS ON THE ROLE OF MITOCHONDRIAL Ca2+ HANDLING AND CYTOSOLIC Ca2+ BUFFERS. J Biol Chem 290: 28214-28230.
4. Faas GC, Schwaller B, Vergara JL, Mody I (2007) Resolving the fast kinetics of cooperative binding: Ca2+ buffering by calretinin. PLoS Biol 5: e311.
