# Supplementary material for: The Effect of Gap Junctional Coupling on the Spatiotemporal Patterns of Ca2+ Signals and the Harmonization of Ca2+-Related Cellular Responses
Source: PLoS Comput Biol. 2016 Dec 27;12(12):e1005295. doi: 10.1371/journal.pcbi.1005295 (PMC5226819; doi:10.1371/journal.pcbi.1005295)
Supplement: S3 Text — (PDF) [file pcbi.1005295.s023.pdf]

Supplementary simulations for testing the robustness of the model

In this part we aimed to demonstrate that the observed phenomena reported in the Main Text do not critically depend on certain assumptions and/or parameters used in our modeling.

1. Saturating J_{SERCA} and J_{EFF}

Assuming that the Ca^{2+} influx into the ER (J_{SERCA}) saturates at high cytosolic Ca^{2+} concentration, then equation (12) can be replaced with the following Hill-equation based on the cytosolic Ca^{2+}-concentration x:

$$J_{SERCA}(x) = V_{s,max} \frac{x^{h_s}}{K_s^{h_s} + x^{h_s}} \tag{1}$$

with the following parameters : $V_{s,max} = 170$ (nM/s), $K_s = 480$ (nM) and $h_s = 2.4$. Similarly, we can postulate that J_{EFF} saturates at high cytosolic Ca^{2+} concentration; consequently, equation (7) from the Main Text can be replaced by

$$J_{EFF}(x) = V_{e,max} \frac{x^{h_e}}{K_e^{h_e} + x^{h_e}} \tag{2}$$

with $V_{e,max} = 260$ (nM/s), $K_e = 460$ (nM) and $h_e = 3.5$. Under these conditions no qualitative changes were observed as shown in Fig. A.

2. Driving force

Next we analyzed the effect of the driving force. Ca^{2+} fluxes, either through leak channels or via $InsP_3R$ depends on the driving force originating from an electrochemical gradient. This gradient consists of two parts, the chemical gradient, i.e. the difference in solute concentrations across the membrane, and the electrical gradient, i.e. the difference in charge across the membrane. The driving force can be calculated from the following function based on the cytosolic and luminal Ca^{2+} concentrations x and y:

$$G_f(x, y) = R \cdot T \cdot \ln\left(\frac{y}{x}\right) + Z \cdot F \cdot V_m \tag{3}$$

In this equation, R represents the gas constant, T the absolute temperature, Z is the ionic charge, F the Faraday constant, and V_m is the membrane potential. Of note, currently nothing is known about changes of the ER membrane potential during Ca^{2+} oscillations. Considering that carbonyl cyanide m-chlorophenyl hydrazine (CCCP), a proton uncoupler, does not evoke ER Ca^{2+} store depletion, we assume that the ER membrane potential, i.e. the potential difference between the luminal and cytosolic part of the membrane must be close to zero. In this case G_f is linearly correlated with $\ln(y/x)$, i.e.

$$G_f(x, y) = r_g \cdot \ln\left(\frac{y}{x}\right), \tag{4}$$

with r_g representing the proportionality constant. Incorporating it in our model by replacing equations (13) and (16) by

$$J_{ERLEAK} = G_f(x,y) \cdot \beta \tag{5}$$

$$J_{EREFF}(x,y,v,t) = G_f(x,y) \cdot \left(J_{cytdep}(x,v,t) + J_{ERdep}(y,v,t)\right) \mathbf{1}_{\{J_{cytdep}(x,v,t)+J_{ERdep}(y,v,t)>0\}} \tag{6}$$

with β, J_{cytdep} and J_{ERdep} defined in the Main Text leads to the following results illustrated in Fig. B.

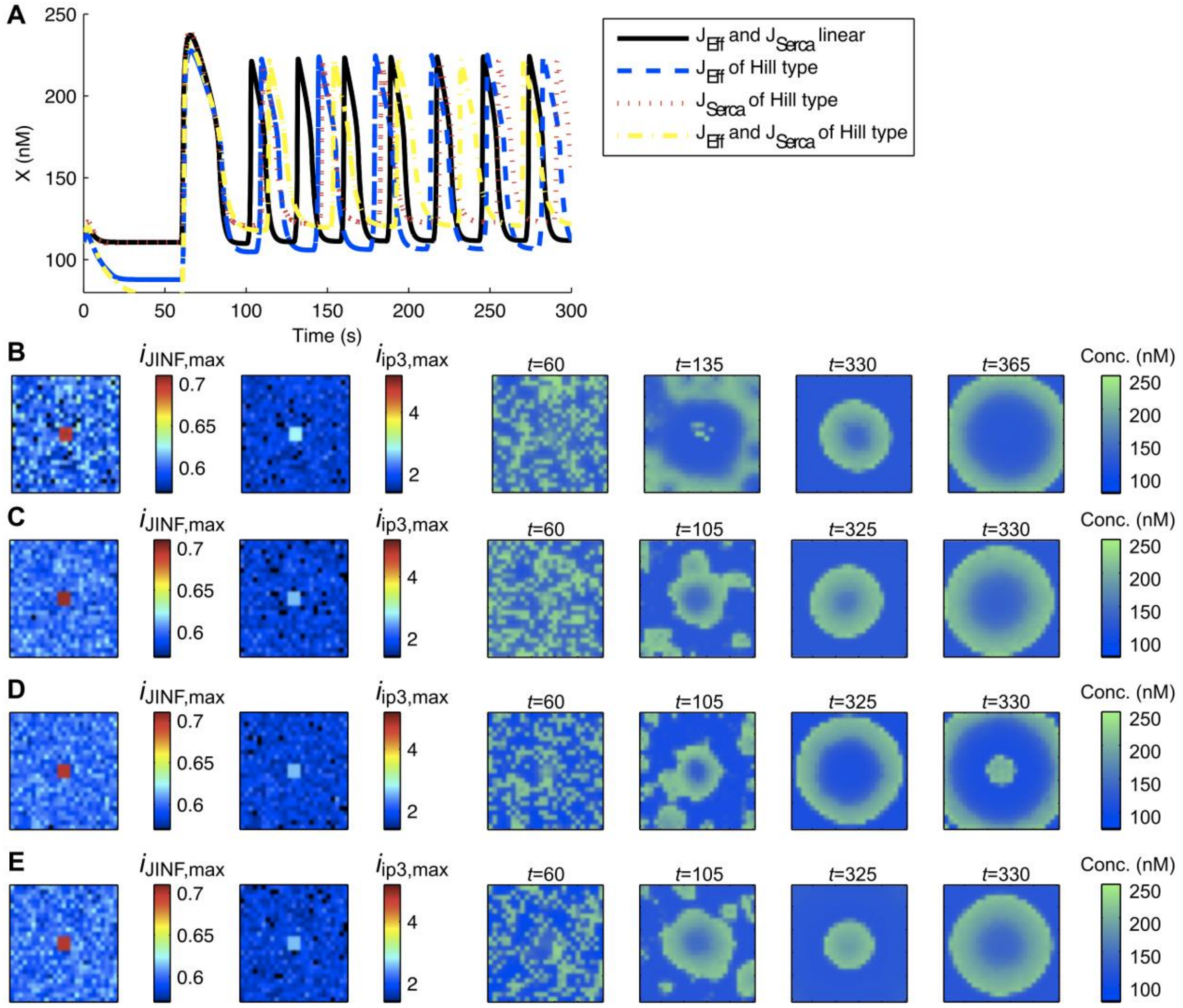

Fig A. Effect of saturating fluxes of J_{SERCA} and J_{EFF}. (A) In a one-cell model ($n = m = 1$), the cytosolic Ca^{2+} concentration oscillates in a similar manner as when applying equation (12) for $J_{SERCA}(x)$ and/or (7) for $J_{EFF}(x)$ as detailed in the Main Text. (B) The Figure contains the same panel as is shown in Fig. 9B of the Main Text: a more sensitive central zone enables concentric circular waves to appear in the random model with low noise and moderate gap-junctional coupling. (C) The same framework as in (B) is shown, however equation (12) from the Main Text is replaced by the Hill curve (1) defined in this Supplementary Text. (D) The same framework as in (B), but equation (7) from the Main Text is replaced by the Hill curve described in equation (2). (E) The equation (12) from the Main Text is replaced by (1) and equation (7) from Main Text by (2). In all conditions, the general behavior, i.e. the generation of concentric Ca^{2+} oscillations is qualitatively similar as what is observed in panel (B).

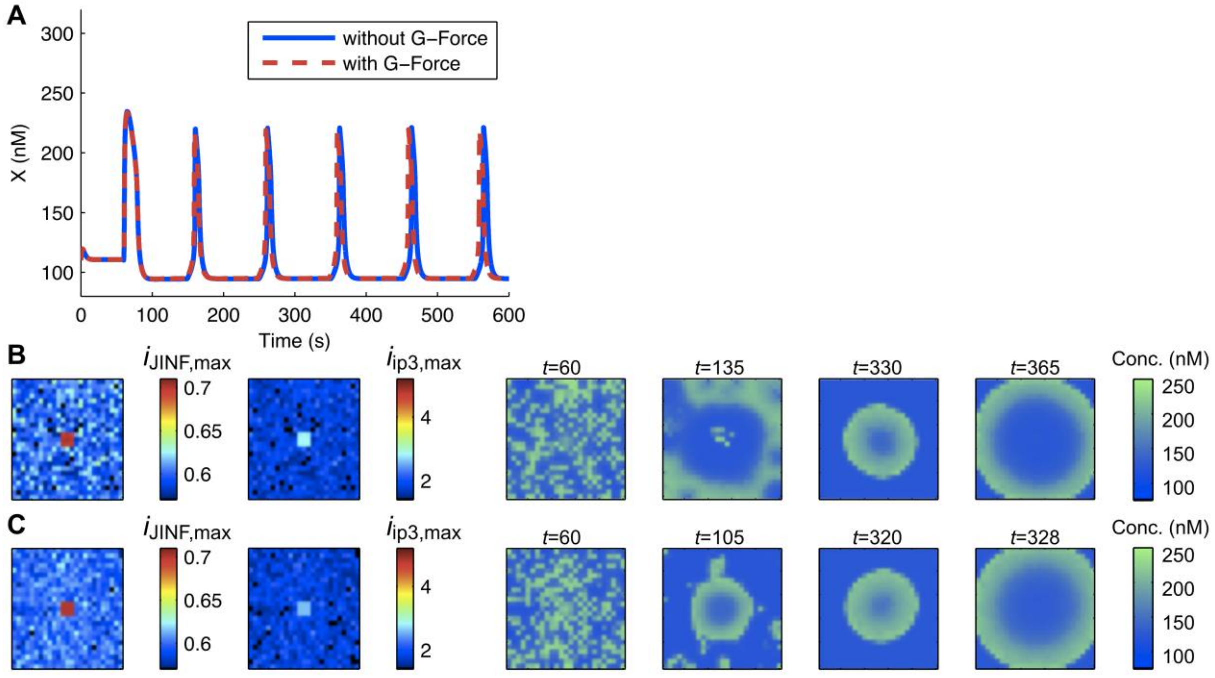

Fig B. Effect of the driving force. (A) In a one-cell model ($n = m = 1$), the cytosolic Ca^{2+} concentration still oscillates in a similar manner irrespective of taking G_f (equation (4)) into account. For this simulation $i_{JINF,max} = 0.6$. (B) For comparison, Fig. 9B from the Main Text is depicted: a more sensitive central zone enables concentric circular waves to appear in the random model with low noise and moderate gap-junctional coupling. (C) The same framework and parameters were used as in (B), but the equation G_f is incorporated with equations (5) and (6). Again, the overall behavior of the oscillations is qualitatively similar as what is observed in (B).

3. Use of the binding and dissociation rate constants of Ca^{2+} for the inhibitory and activatory binding sites of $InsP_3R$

Instead of considering a steady-state $InsP_3R$ model, we completed our model considering the rate constants of the activating/inhibitory Ca^{2+}-binding sites of $InsP_3R$. As there is no experimental data on the precise values of binding and dissociation rate constants of Ca^{2+} to $InsP_3R$, few suppositions were required. The assumption that the activating Ca^{2+}-binding site has faster rate constants than the inhibitory binding site is often made to generate Ca^{2+} oscillations in different models. The prototype of this kind of Ca^{2+} models is the De Young-Keizer model [1]. This model and several other derivatives are very efficient for the simulation of fast sinusoidal oscillations, but fail to recapitulate the slow baseline spiking oscillations. Sinusoidal oscillation is a term for a continuous fluctuation in the cytoplasmic Ca^{2+} concentration (c_{cyt}) starting from a c_{cyt} value that is elevated compared to the resting c_{cyt}. The baseline spiking oscillations represent discrete Ca^{2+} transients arising from a constant basal c_{cyt} level [2]. A further disadvantage of the De Young-Keizer model is that only the actual [$InsP_3$] is considered as the frequency-determining factor. Thus, in contradiction with the five phases experimentally observed during a Ca^{2+}-oscillation cycle (interspike, foot, fast upstroke, shoulder, slow recovery [3]) it contains only two phases; an ascending and a descending phase. Our model does not use the concept of rate constants of the activating/inhibitory Ca^{2+}-binding sites of $InsP_3R$ to generate Ca^{2+} oscillations. Since different modeling concepts are not exclusive, it is very likely that in reality in biological systems, cells exploit several mechanisms at the same time for the generation of Ca^{2+} oscillations. Here we demonstrate that De Young-Keizer (DYK) $InsP_3R$ model can be incorporated

into ours. Hence, we are able to create a model containing an additional factor for the generation of Ca^{2+} oscillations.

The DYK model assumes that the $InsP_3R$ is composed of independent, however identical subunits. Each of the subunits includes a binding site for activating $InsP_3$, activating Ca^{2+} and inactivating Ca^{2+}. Only binding of $InsP_3$ to the $InsP_3$-activating site and binding of Ca^{2+} to the Ca^{2+}-activating site leads to a Ca^{2+} flux through the receptor. Each state of the subunit is given by x_{ijk}, and $i, j, k \in [0,1]$, where the first index refers to the $InsP_3$-binding site, the second to the Ca^{2+}-activation site, and the third to the Ca^{2+}-inactivation site. If any of the indices i, j or k are equal to 1, the binding site is occupied; otherwise the binding site is unoccupied. The model generates eight possible receptor states, with correlated transitions between them. However, if we assume, in agreement with the DYK model that $InsP_3$ binding is very fast, then the model can be reduced to a four-state model. Each state of the subunit is given by S_{jk} and $j, k \in [0,1]$, where the first index refers to the Ca^{2+}-activation site, and the second to the Ca^{2+}-inactivation site (See Fig. C).

The model assumes that the $InsP_3R$ passes a Ca^{2+} current only when subunits are in the state S_{10}. The four states of the DYK model consist of three differential equations for the four states (with the constraint that $S_{10} + S_{11} + S_{00} + S_{01} = 1$, due to the fact that S_{jk} are probabilities). We slightly modified this model and the dissociation and association rate constants to be joined with our model. The differential equations for the receptor states are based on mass-action kinetics, and we denote $S_{jk,v}(t)$, the state j, k of cell v at time t. In the following system of differential equations we removed the dependences on t to simplify the notations. All parameters are shown in Table A.

$$\begin{cases} \frac{dS_{00,v}}{dt} = -(k_{p4} + k_{p5})X_v \cdot S_{00,v} + k_{m4} \cdot S_{01,v} + k_{m5} \cdot S_{10,v} \\ \frac{dS_{01,v}}{dt} = -\left(k_{m4} + k_{p5}X_v\right) \cdot S_{01,v} + k_{p4}X_v \cdot S_{00,v} + k_{m5} \cdot S_{11,v} \\ \frac{dS_{10,v}}{dt} = -(k_{p4}X_v + k_{m5}) \cdot S_{10,v} + k_{p5}X_v \cdot S_{00,v} + k_{m4} \cdot S_{11,v} \end{cases} \quad (7)$$

together with the condition

$$S_{11,v} = 1 - (S_{00,v} + S_{01,v} + S_{10,v}) \quad (8)$$

so that we replace equation (14) from the Main Text by the following function that is dependent on the cytosolic Ca^{2+} concentration x at time t, in line with the DYK model [1],

$$J_{cytdep}(x, v, t) = r_{DYK,1}\left(S_{10,v} \cdot InsP_3(v,t)\right)^3 + r_{DYK,2} \quad (9)$$

	Parameter name	Value
Constants	b_4	0.066 /(μM · s)
	b_5	0.66 /(μM · s)
	a_4	0.0096 /s
	a_5	0.55 /s
	$r_{DYK,1}$	15000 nM/s
	$r_{DYK,2}$	−1700 nM/s

Table A. Parameters used in the De Young-Keizer (DYK) model.

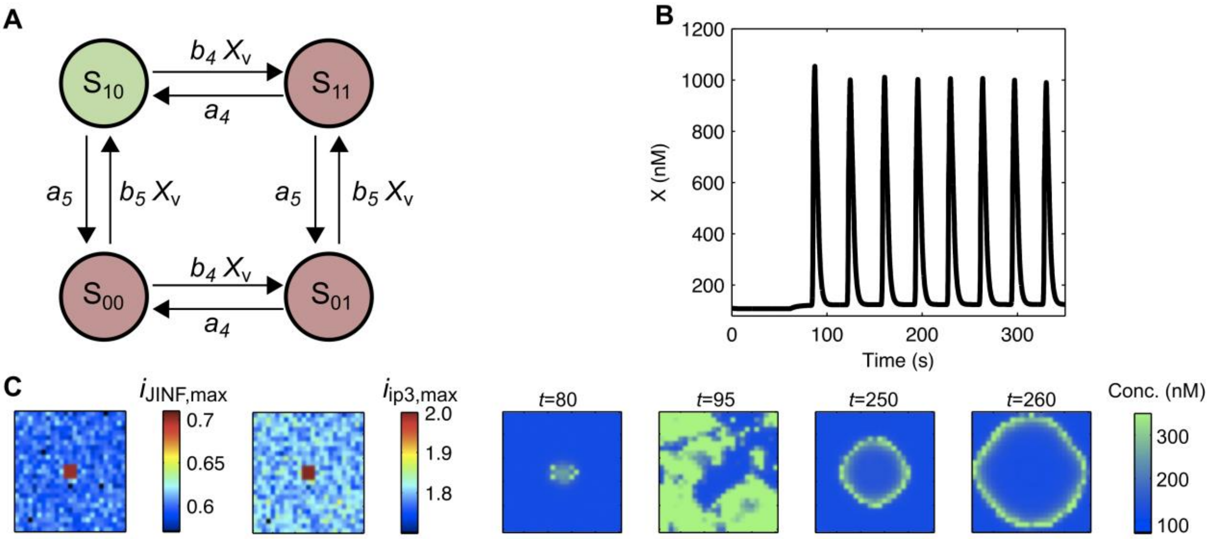

Fig C. De Young-Keizer (DYK) model. (A) The transition scheme according to the DYK model containing four binding sites is shown (see Table A for the parameter values). (B) In a one-cell model ($n = m = 1$), the cytosolic Ca^{2+} concentration still oscillates in a similar manner as shown in Fig. 2 of the Main Text. Note that the oscillations shown here have higher $c_{cyt,max}$ values than the ones shown in Fig. 9B of the Main Text. The parameters are the ones reported in supplemental Table A and Table 1 in the Main Text with $i_{JINF,max} = 0.7$ and $i_{IP3,max} = 2$. (C) A similar framework as used in Fig. 9B from the Main Text is shown: a more sensitive central zone enables concentric circular waves to appear in a random model with low noise and moderate gap-junctional coupling. Since this mixed model is more sensitive, the parameters have been slightly adapted with $i_{JINF,max,1} = 0.7$ and $i_{IP3,max,1} = 2$ in the central (sensitive) zone and $i_{JINF,max,0} = 0.6$ and $i_{IP3,max,0} = 1.8$ otherwise. The noise for [$InsP_3$] has also been slightly reduced to $\sigma_{i_{IP3,max}} = 0.025$.

This mixed model is able to generate Ca^{2+} oscillations. The oscillation frequency is positively correlated with [$InsP_3$] and with $J_{inf,max}$ values. Ca^{2+} ions passing through gap junctions are able to synchronize the Ca^{2+} oscillations forming Ca^{2+} phase waves. However the system is more sensitive to the changes in [$InsP_3$] and $J_{inf,max}$. As a result, we needed to select slightly different initial parameters. Nevertheless, we observed no qualitative changes in the Ca^{2+} phase wave phenomena as shown in Fig. 3 of the Main Text.

In conclusion, synchronization can be produced by small amounts of Ca^{2+} diffusion through gap junctions via phase wave phenomena. The critical point is to employ a model in which the frequency of the Ca^{2+} oscillations is regulated not only by the prevailing [$InsP_3$], but also by the Ca^{2+} handling. If the model presumes that the steady-state concentration of $InsP_3$ is the only factor determining the oscillations frequency, then evidently, only $InsP_3$ may serve as the coupling agent [4]. Nonetheless, other factors than the actual [$InsP_3$] influence the Ca^{2+} oscillations frequency. There are many reports demonstrating that the Ca^{2+} influx across the plasma membrane influences the oscillation frequency [3,5-7] and moreover that also mitochondria's Ca^{2+} transport function plays a role [8,9].

References

1. De Young GW, Keizer J (1992) A single-pool inositol 1,4,5-trisphosphate-receptor-based model for agonist-stimulated oscillations in Ca2+ concentration. Proceedings of the National Academy of Sciences of the United States of America 89: 9895-9899.

2. Berridge MJ (1990) Calcium oscillations. J Biol Chem 265: 9583-9586.
3. Pecze L, Schwaller B (2015) Characterization and modeling of Ca(2+) oscillations in mouse primary mesothelial cells. Biochim Biophys Acta 1854: 632-645.
4. Dupont G, Tordjmann T, Clair C, Swillens S, Claret M, et al. (2000) Mechanism of receptor-oriented intercellular calcium wave propagation in hepatocytes. FASEB J 14: 279-289.
5. Hashitani H, Yanai Y, Suzuki H (2004) Role of interstitial cells and gap junctions in the transmission of spontaneous Ca2+ signals in detrusor smooth muscles of the guinea-pig urinary bladder. J Physiol 559: 567-581.
6. Hennig GW, Smith CB, O'Shea DM, Smith TK (2002) Patterns of intracellular and intercellular Ca2+ waves in the longitudinal muscle layer of the murine large intestine in vitro. J Physiol 543: 233-253.
7. Berridge MJ (1992) Inositol trisphosphate and calcium oscillations. Adv Second Messenger Phosphoprotein Res 26: 211-223.
8. Pecze L, Blum W, Schwaller B (2015) Routes of Ca2+ Shuttling during Ca2+ Oscillations: FOCUS ON THE ROLE OF MITOCHONDRIAL Ca2+ HANDLING AND CYTOSOLIC Ca2+ BUFFERS. J Biol Chem 290: 28214-28230.
9. Wacquier B, Combettes L, Van Nhieu GT, Dupont G (2016) Interplay Between Intracellular Ca(2+) Oscillations and Ca(2+)-stimulated Mitochondrial Metabolism. Sci Rep 6: 19316.
